# Supplementary material for: Co-designing organisational improvements and interventions to increase inpatient activity in four stroke units in England: a mixed-methods process evaluation using normalisation process theory
Source: BMJ Open. 2021 Jan 26;11(1):e042723. doi: 10.1136/bmjopen-2020-042723 (PMC7839845; doi:10.1136/bmjopen-2020-042723)
Supplement: Supplementary data [file bmjopen-2020-042723supp001.pdf]

Supplementary File 1: Data collection linked to Normalisation Process Theory

**Focus for and use of Normalisation Process Theory in the process evaluation of the CREATE study. NB: the elements are not necessarily progressed through in a linear way; there is some degree of cyclical movement back and forth between elements.**			
**NPT element**	**Focus**	**Data collection (keep in NVivo or as word documents)**	**Notes**
**Coherence**	EBCD/AEBCD training for staff from sites	Researcher reflective notes on the training days<br><br>Point of Care Foundation training feedback sheets (anonymised) were available for the full day training and then feedback/post it notes from the half day training.	
	Informal staff comments and observations during: non-participant observations	Researchers' reflective notes on staff perceptions and understanding of the aims of the study (increase patient activity outside of therapy through co-designed interventions.	Include any evidence of access to the Kings Fund Toolkit, any evidence of dialogue between stroke unit teams (e.g. in handovers, at MDT or other staff meetings)
	Staff interviews pre interventions with:<br>a) EBCD/AEBCD champions at each site<br>b) Remaining staff interviewees	Interview transcripts (where these occurred)<br><br>Researchers' reflective notes on staff perceptions and understanding of the aims of the study and any evidence of actions to involve the wider stroke unit team<br>Researchers' reflective notes on staff perceptions and understanding of the aims of the study<br>Review documentary evidence (notes of meetings/action plans/reviews of action plans)	(Overlaps with cognitive participation, collective action and reflexive monitoring)
	Study Oversight Groups (SoG) meetings	SoG members at sites include: Deputy Directorate Manager, Therapy Lead (Manager), Service Improvement Lead, Volunteer Manager, Stroke Specialist Nurse.	These were largely virtual meetings - review notes and email trails/responses from any SoG members

Clarke D, *et al. BMJ Open* 2021; 11:e042723. doi: 10.1136/bmjopen-2020-042723

Supplementary File 1: Data collection linked to Normalisation Process Theory

**Cognitive participation**	Staff interviews post interventions with:<br>a) EBCD/AEBCD champions at each site<br>b) Remaining staff interviewees	Interview transcripts<br><br>Researchers' reflective notes on staff perceptions and understanding of the aims of the study and any evidence of actions to involve the wider stroke unit team	***Overlaps with coherence***
	Preparation for and conduct of staff event and patient event and joint event	Patient and staff questionnaire* feedback<br>EBCD champions individual reflective summary (Guide given)<br>Researchers' reflective notes on staff perceptions and understanding of the aims of the study and any evidence of actions to involve the wider stroke unit team	Questionnaires and reflective summary sheets adapted from those used in the Locock et al (2014) study
	Co-design group meetings	Patient and staff questionnaire feedback<br>EBCD champions individual reflective summary<br>Review documentary evidence (notes of meetings/action plans/reviews of action plans)<br>Researchers' reflective notes on staff perceptions and understanding of the co-design work and any evidence of actions to involve the wider stroke unit team.	As above- seek questionnaires and reflective summaries after each event or meeting.
	Study Oversight Groups (SoG) any ongoing meetings	Researchers' reflective notes on staff perceptions and understanding of the progress of the study and of staff and patient involvement in this. Review documentary evidence (notes of meetings/action plans/reviews of action plans)	

Clarke D, *et al. BMJ Open* 2021; 11:e042723. doi: 10.1136/bmjopen-2020-042723

Supplementary File 1: Data collection linked to Normalisation Process Theory

**Collective action**	Co-design groups<br>And implementation activity<br><br>And feedback from the celebratory events at both sites	Any patient and staff questionnaire* feedback<br>EBCD champions individual reflective summary<br>Review documentary evidence (notes of meetings/action plans/reviews of action plans)<br><br>Researchers' reflective notes on staff's and patients' and carers' engagement with the co-design work and evidence of actions to involve the wider stroke unit team. (E.g. be alert to discussion at MDT or other meetings), evidence of impacts on existing work of staff and how this is managed/discussed.<br><br>Researchers' reflective notes on range of interventions/activities introduced and how these are managed by individuals and groups (e.g. therapists/nurses/volunteers) and whether and how these are publicised by the EBCD champions or others	
**Reflexive monitoring**	Co-design groups<br>And implementation activity<br><br>Staff interviews with:<br>a) EBCD/AEBCD champions at each site<br>b) Remaining staff<br><br>Patient and carer interviews<br><br>Informal staff comments and observations during: non-participant observations	Data collection as above<br><br>Interview transcripts<br><br>Researchers' reflective notes on staff perceptions of the study and how the co-design work and interventions have affected patients' activity levels and overall inpatient experience.<br>Review the involvement of the wider stroke unit team in interventions to increase activity levels<br><br>Review documentary evidence (notes of meetings/action plans/reviews of action plans)<br><br>Interview transcripts<br><br>Researchers' reflective notes on staff perceptions and understanding of the outcomes of the study (increase patient activity outside of therapy through co-designed interventions.	**Links back to Cognitive participation**- review reflexive monitoring activity at the when co-design groups are established and as interventions/activities are piloted

Supplementary File 1: Data collection linked to Normalisation Process Theory

**Additional focus on:**	Absence of separate staff and patient meetings in AEBCD sites<br><br>Shortened timescale for co-design at AEBCD sites<br><br>Differences in level of patient and carer recruitment for EBCD/AEBCD groups between sites.	Consider the impact on the joint meeting and progression to co-design work<br><br>Examples are numbers of people involved different sites.	
**Organisational factors impacting on set up of CREATE at sites:**	Consider: managerial involvement or its absence, project disruption at sites interest and support at the wider organisational level or beyond	Consider other organisational factors relevant to the conduct of the study at each site including:<br><br>Key staff absence or key staff leaving the organisation<br><br>Ongoing or specific staffing problems<br><br>Positive/innovative factors such as the fundraising and design competition at site 3.<br><br>And the pre-existing funding for day room refurbishment at Site 2, and the ongoing Rooting and Fruiting gardening project at Site 4	

Clarke D, *et al. BMJ Open* 2021; 11:e042723. doi: 10.1136/bmjopen-2020-042723
