# Supplementary material for: Co-designing organisational improvements and interventions to increase inpatient activity in four stroke units in England: a mixed-methods process evaluation using normalisation process theory
Source: BMJ Open. 2021 Jan 26;11(1):e042723. doi: 10.1136/bmjopen-2020-042723 (PMC7839845; doi:10.1136/bmjopen-2020-042723)
Supplement: Supplementary data [file bmjopen-2020-042723supp002.pdf]

Supplementary file 2: Demographic data all participants

Patient demographics

**Site 1 Patient interviews (n= 9) pre-implementation of EBCD**	
**Age**	**Gender**
65 +/-12 years	M = 5<br>F = 4
**Site 1 Patient interviews (n= 5) post-implementation of EBCD**	
65 +/-13 years	M = 3<br>F = 2
**Site 2 Patient interviews (n= 9) pre-implementation of EBCD**	
68 =/- 18 years	M= 5<br>F= 4
**Site 2 Patient interviews (n=6) post-implementation of EBCD**	
73 +/- 12 years	M =6
**Site 3 Patient interviews (n= 9) pre-implementation of EBCD**	
61 +/- 16 years	M = 5<br>F = 4
**Site 3 Patient interviews (n= 6) post-implementation of EBCD**	
67 +/- 18 years	M = 4<br>F = 2
**Site 4 Patient interviews (n= 4) pre-implementation of EBCD**	
72 +/- 7 years	M = 3<br>F = 1
**Site 4 Patient interviews (n= 6) post-implementation of EBCD**	
68 +/- 10 years	M = 2<br>F = 4

F female, M male

Family carer demographics

**Site 1 Carer interviews (n= 4) pre-implementation of EBCD**	
Age	Gender
45-54	F
18-24	M
55-64	M
55-64	F
**Site 1 Carer interviews (n= 5) post-implementation of EBCD**	
18-24	M
55-64	M
45-54	F
65-74	M
75-84	F

Site 2 Carer interviews (n=4) pre-implementation of EBCD	
55-64	F
65-74	F
65-74	F
75-84	M
**Site 2 Carer interviews (n= 2) post-implementation of EBCD**	
75-84	F
75-84	F
**Site 3 Carer interviews (n=3) pre-implementation of AEBCD**	
65-74	F
55-64	F
75-84	F
**Site 3 Carer interviews (n=3) post-implementation of AEBCD**	
65-74	F
65-74	M
18-24	F
**Site 4 Carer interviews (n=2) pre-implementation of AEBCD**	
55-64	F
65-74	M
**Site 4 Carer interviews (n=5) post-implementation of AEBCD**	
85-94	M
65-74	M
55-64	M
65-74	M
55-64	F

Demographic data staff participants

Site 1 Staff interviews (n=13) pre-implementation of EBCD	
**Age**	**Profession**
45-54	Doctor
25-34	RN
35-44	RN
35-44	HCSW
45-54	HCSW
45-54	Domestic
35-44	OT
25-34	OT
45-54	PT
25-34	PT
35-44	Clinical Psychologist
25-34	SALT
35-44	Dietician
**Site 1 Staff interviews (n=8) post-implementation of EBCD**	
25-34	SALT
25-34	PT
35-44	OT
35-44	Dietician
35-44	SRA
35-44	Operations manager
25-34	RN
45-54	Consultant
**Site 2 Staff interviews (n=15) pre-implementation of EBCD**	
35-44	PT
25-34	PT
18-24	SRA
25-34	HCSW

35-44	SALT
35-44	OT
25-34	Dietician
25-34	OT
25-34	RN
25-34	RN
25-34	RN
55-64	Volunteer
25-34	Dietician
35-44	HCSW
35-44	Doctor
**Site 2 staff interviews (n= 7) post-implementation of EBCD**	
25-34	RN
25-34	PT
35-44	HCA
25-34	SRA
25-34	RN
25-34	OT
45-54	HCSW
**Site 3 Staff interviews (n= 6) pre-implementation of AEBCD**	
35-44	Clinical Psychologist
35-44	OT
25-34	PT
35-44	RN
45-54	PT
45-54	Doctor
**Site 3 Staff interviews (n= 8) post-implementation of AEBCD**	
35-44	Clinical Psychologist
25-34	OT
25-34	PT
45-54	RN
45-54	PT
45-54	Doctor
35-44	HCSW
35-44	SRA
**Site 4 Staff interviews (n= 7) pre-implementation of AEBCD**	
35-44	TA
35-44	Doctor
45-54	RN
25-34	PT
25-34	OT
55-64	RN
45-54	SALT
**Site 4 Staff interviews (n= 9) post-implementation of AEBCD**	
25-34	SALT
35-44	RN
25-34	HCSW
25-34	PT
55-64	RN
45-54	RN
25-34	OT
45-54	TA
35-44	RN

PT physiotherapist, OT, occupational therapist, SALT speech and language therapist, RN, registered nurse, SRA stroke rehabilitation assistant, TA therapy assistant, HCSW healthcare support worker.
