# Supplementary material for: Co-designing organisational improvements and interventions to increase inpatient activity in four stroke units in England: a mixed-methods process evaluation using normalisation process theory
Source: BMJ Open. 2021 Jan 26;11(1):e042723. doi: 10.1136/bmjopen-2020-042723 (PMC7839845; doi:10.1136/bmjopen-2020-042723)
Supplement: Supplementary data [file bmjopen-2020-042723supp003.pdf]

Supplementary file 3: Number of Co-design groups held

	**Co-design participants**			**Number of co-design group meetings**
	**Stroke survivors**	**Carers**	**Staff (includes volunteers)**	
**Site 1 (EBCD)**	5	6	13	10
**Site 2 (EBCD)**	4	4	12	9
**Site 3 (AEBCD)**	11	7	14	15
**Site 4 (AEBCD)**	3	4	15	9
**TOTAL**	**23**	**21**	**54**	**43**
