# Supplementary material for: Co-designing organisational improvements and interventions to increase inpatient activity in four stroke units in England: a mixed-methods process evaluation using normalisation process theory
Source: BMJ Open. 2021 Jan 26;11(1):e042723. doi: 10.1136/bmjopen-2020-042723 (PMC7839845; doi:10.1136/bmjopen-2020-042723)
Supplement: Author's manuscript [file bmjopen-2020-042723.draft_revisions.pdf]

BMJ Open is committed to open peer review. As part of this commitment we make the peer review history of every article we publish publicly available.

When an article is published we post the peer reviewers' comments and the authors' responses online. We also post the versions of the paper that were used during peer review. These are the versions that the peer review comments apply to.

The versions of the paper that follow are the versions that were submitted during the peer review process. They are not the versions of record or the final published versions. They should not be cited or distributed as the published version of this manuscript.

BMJ Open is an open access journal and the full, final, typeset and author-corrected version of record of the manuscript is available on our site with no access controls, subscription charges or pay-per-view fees (http://bmjopen.bmj.com).

If you have any questions on BMJ Open's open peer review process please email info.bmjopen@bmj.com

BMJ Open

Co-designing organisational improvements and interventions to increase inpatient activity in four stroke units in England; a mixed-methods process evaluation using Normalisation Process Theory.

Journal:	*BMJ Open*
Manuscript ID	bmjopen-2020-042723
Article Type:	Original research
Date Submitted by the Author:	14-Jul-2020
Complete List of Authors:	Clarke, David; University of Leeds Faculty of Medicine and Health, Academic Unit of Ageing and Stroke Research, Leeds Institute of Health Sciences<br>Gombert-Waldron, Karolina; Kingston University & St George's, University of London, Faculty of Health and Social Care Sciences<br>Honey, Stephanie; University of Leeds, Leeds Institute of Health Sciences<br>Cloud, Geoffrey; Monash University Melbourne and Alfred Health, Department of Clinical Neuroscience, CCS.<br>Harris, Ruth; King\'s College London<br>Macdonald, Alastair; The Glasgow School of Art<br>McKevitt, Christopher; King's College London, Department of Public Health Sciences<br>Robert, Glenn; King's College London<br>Jones, Fiona; St Georges University of London
Keywords:	Stroke < NEUROLOGY, REHABILITATION MEDICINE, Quality in health care < HEALTH SERVICES ADMINISTRATION & MANAGEMENT, QUALITATIVE RESEARCH

SCHOLARONE™
Manuscripts

I, the Submitting Author has the right to grant and does grant on behalf of all authors of the Work (as defined in the below author licence), an exclusive licence and/or a non-exclusive licence for contributions from authors who are: i) UK Crown employees; ii) where BMJ has agreed a CC-BY licence shall apply, and/or iii) in accordance with the terms applicable for US Federal Government officers or employees acting as part of their official duties; on a worldwide, perpetual, irrevocable, royalty-free basis to BMJ Publishing Group Ltd ("BMJ") its licensees and where the relevant Journal is co-owned by BMJ to the co-owners of the Journal, to publish the Work in this journal and any other BMJ products and to exploit all rights, as set out in our licence.

The Submitting Author accepts and understands that any supply made under these terms is made by BMJ to the Submitting Author unless you are acting as an employee on behalf of your employer or a postgraduate student of an affiliated institution which is paying any applicable article publishing charge ("APC") for Open Access articles. Where the Submitting Author wishes to make the Work available on an Open Access basis (and intends to pay the relevant APC), the terms of reuse of such Open Access shall be governed by a Creative Commons licence – details of these licences and which Creative Commons licence will apply to this Work are set out in our licence referred to above.

Other than as permitted in any relevant BMJ Author's Self Archiving Policies, I confirm this Work has not been accepted for publication elsewhere, is not being considered for publication elsewhere and does not duplicate material already published. I confirm all authors consent to publication of this Work and authorise the granting of this licence.

For peer review only

Title:

Co-designing organisational improvements and interventions to increase inpatient activity in four stroke units in England; a mixed-methods process evaluation using Normalisation Process Theory

Authors:

David J Clarke[1], Karolina Gombert-Waldron[2], Stephanie Honey[1], Geoffrey C Cloud[3], Ruth Harris[4], Alastair Macdonald[5], Christopher McKevitt[6], Glenn Robert[4], Fiona Jones[2].

[1]Leeds Institute of Health Sciences. University of Leeds, UK
[2]Faculty of Health and Social Care Sciences. Kingston University & St George's, University of London. UK.
[3] Department of Clinical Neuroscience, CCS, Monash University Melbourne and Alfred Health, Melbourne, Australia.
[4]Florence Nightingale Faculty of Nursing, Midwifery & Palliative Care, King's College London. UK.
[5]School of Design, Glasgow School of Art. UK.
[6]School of Population Health & Environmental Sciences, Faculty of Life Sciences and Medicine, King's College London. UK.

Corresponding author: **David J Clarke**
Corresponding author contact details:
d.j.clarke@leeds.ac.uk ORCID ID: 0000-0001-6279-1192

Declared competing interests of authors:
GR teaches on the EBCD training courses run by 'The Point of Care Foundation' in London. GR is one of the originators of EBCD.

Word count: 6855

For peer review only

Abstract

Objective: To explore facilitators and barriers to using Experience-based Co-design (EBCD) and accelerated EBCD (AEBCD) in the development and implementation of interventions to increase activity opportunities for inpatient stroke survivors.

Design: Mixed-methods process evaluation underpinned by normalisation process theory (NPT).

Setting: Four post-acute rehabilitation stroke units in England.

Participants: Stroke survivors, family members, stroke unit staff, hospital managers, support staff and volunteers. Data informing our NPT analysis comprised: ethnographic observations, n= 366 hours; semi-structured interviews with 76 staff, 53 stroke survivors and 27 family members pre or post EBCD/AEBCD implementation; and observation of 43 co-design meetings involving 23 stroke survivors, 21 family carers and 54 staff

Results: Former patients and families valued participation in EBCD/AEBCD perceiving they were equal partners in co-design. Staff engaged with EBCD/AEBCD, reporting it as valuable improvement approach leading to increased activity opportunities. The structured EBCD/AEBCD approach was influential in enabling coherence and cognitive participation and legitimated staff involvement in the change process. Researcher facilitation of EBCD/AEBCD supported cognitive participation, collective action and reflexive monitoring; these were important in implementing and sustaining co-design activities. Observations and interviews post EBCD/AEBCD cycles confirmed creation and use of new social spaces and increased activity opportunities in all units. Changes occurred without increased staffing or additional organisational expenditure. EBCD/AEBCD facilitated engagement with wider hospital resources and local communities, further enhancing activity opportunities. However, outside of structured group activity many individual staff-patient interactions remained task-focused.

Conclusions: EBCD/AEBCD facilitated the development and implementation of environmental changes and revisions to work routines which supported increased activity opportunities in stroke units providing post-acute and rehabilitation care. Former stroke patients and carers contributed to improvements. NPT's generative mechanisms were instrumental in analysis and interpretation of facilitators and barriers at the individual, group and organisational levels and can help inform future implementations of similar approaches.

Strengths and limitations of this study:

- This process evaluation reports the first use of Experience-based Co-design (EBCD) and accelerated EBCD (AEBCD) in stroke services.
- Analysis informed by normalisation process theory before, during and after use of EBCD/AEBCD provided for an in-depth understanding of staff engagement, local organisational contexts, the impact of co-designed changes on day-to-day working practices of stroke unit staff and the experiences of stroke survivors both as inpatients and as participants in EBCD/AEBCD.
- Recruitment of stroke survivors and family members to participation in EBCD/AEBCD activities was good across all sites but it proved more difficult to recruit former inpatient stroke survivors to participate in post EBCD/AEBCD evaluation interviews.
- The process evaluation was not designed to generate data to evaluate the longer-term sustainability of interventions developed to increase activity opportunities for inpatient stroke survivors.

For peer review only - http://bmjopen.bmj.com/site/about/guidelines.xhtml

- Researchers undertaking the process evaluation were part of the core research team for the CREATE study and not a separately employed process evaluation team.

Introduction

Stroke is the second most common cause of death worldwide[1] and is associated with significant long-term disability[2]. Specialist rehabilitation in stroke units contributes substantially to regaining independence[3]. However, observational studies of patients in stroke units identify high levels of inactivity. In studies spanning forty years, patients have been reported to be physically active between 13% and 23% of the waking day, and engaged in cognitive or social activity between 4% and 32% of the waking day[4-10]. In these studies, activity related largely to participation in planned therapy, which usually includes physiotherapy (PT), occupational therapy (OT), speech and language therapy (SLT)[11-14]. Outside of planned therapy patients report being bored and can be alone and inactive for 60% of the day[7,15-16].

This seemingly intractable and universal problem was addressed by Ellul et al through individualised (physical) activity programmes facilitated by nurses, scheduled social group activities and staff acting as activity leads[17].Similarly, Newell et al introduced regular leisure activity service visits; access to computers and weekly discussion groups, communal lunches and group meetings with other stroke survivors, and encouraged family involvement in therapy practice[18]. Neither study led to wide scale adoption of the approaches reported. Recently, there has been interest in the potential of environmental enrichment (EE)[8,10,19-22] in stroke units. EE is defined as *'interventions designed to facilitate physical (motor and sensory), cognitive and social activity by provision of equipment and organisation of a stimulating environment'* [20]p48. There is also interest in the impact of the built environment on activity and social interaction[23-24] . However, across these studies, there remains limited evidence of sustained change in inpatient activity [19-21].

To date, approaches to address inactivity post-stroke have been largely externally designed and researcher led with limited or no involvement of stroke survivors, caregivers or staff in study design or delivery. An alternative improvement approach used in other healthcare settings involves directly engaging former patients and providers in a collaborative process to 'co-produce' a service that addresses the needs and wants of patients whilst ensuring the improved service can be delivered utilising existing resources[25-28]. Co-production approaches provide a means for stakeholder voices not only to be heard in terms of improving services but can also provide a framework for stakeholder participation throughout the research or improvement process[26,29-31]. Specific forms of co-production

For peer review only - http://bmjopen.bmj.com/site/about/guidelines.xhtml

such as Experience-based Co-design (EBCD)[26,32-33] have been developed and applied in healthcare. The Collaborative Rehabilitation Environments in Acute Stroke (CREATE) study evaluated the impact of using the six-stage EBCD approach in two stroke units and an accelerated version (AEBCD)[34] in two further units (Figure 1) to increase patients' social, cognitive and physical activity. Detailed findings from the CREATE study are reported elsewhere[35]. In summary, qualitative findings indicated it was feasible to co-produce changes in all four stroke units to increase opportunities for social, cognitive and physical activity through joint work in three priority areas: 'Space' (environment), 'Activity' and 'Communication'. Patients, families and staff perceived positive benefits from participating in EBCD/AEBCD. However, quantitative data did not demonstrate consistent increases in physical, social or cognitive activity.

Despite the extensive use of EBCD internationally in the last ten years, there has been limited evaluation of the process and outcomes of the approach, and none in stroke[33,36]. The use of EBCD/AEBCD in the CREATE study as a structured approach through which to develop and implement quality improvments is typical of a complex intervention involving '*multiple components which interact to produce change*'[37]. Medical Research Council[37-38] guidance for the evaluation of complex interventions recommends use of process evaluations to explore not only '*whether interventions 'worked' but [also] how they were implemented, their causal mechanisms and how effects differed from one context to another*'[38]. This paper reports on the embedded process evaluation in the CREATE study. The aim of the process evaluation was to explore facilitators and barriers to using EBCD and AEBCD to develop and implement interventions to increase activity opportunities for inpatient stroke survivors.

Insert Figure 1: *EBCD/AEBCD approach about here*

Methods:

Public and Patient Involvement statement:

Listening to and utilising patients' and carers' voices, experiences and ideas is central to EBCD[26] but patients and the public were also involved from study inception, participating in development of the research proposal which was discussed with stroke survivors and carers at two Stroke Research group meetings. There was strong support for the research, particularly the participatory approach planned; these groups provided important insights about how to facilitate participants' involvement including

For peer review only - http://bmjopen.bmj.com/site/about/guidelines.xhtml

being aware of post-stroke fatigue, challenges of access to hospitals sites, running EBCD/AEBCD events on or near to stroke units and ensuring transport, parking, access and expenses for involvement were considered in advance of onsite participation. A younger stroke survivor and carer became members of the Study Steering Committee (SSC), participating in review of participant information, discussions about conducting observations, interviews and co-design meetings with patients and staff. They helped shape the messaging in EBCD/AEBCD feedback events, reviewed emerging findings and commented on researchers' summary explanations of findings.

CREATE study design and methods

CREATE used a mixed-methods case-comparison design with embedded process evaluation. The wider study methods and findings are detailed elsewhere[35] and only outlined here. EBCD[26,28,33] or AEBCD[34] was introduced into four stroke units (sites 1-4), two in London and two in the North of England (Yorkshire). Nominated staff from each unit were trained in EBCD/AEBCD; researchers facilitated the EBCD/AEBCD process in each site. Sites 1 and 2 completed full EBCD cycles, over 9 months. Sites 3 and 4 completed AEBCD cycles over 6 months (Figure 1). In all sites co-design groups made up of former patients, carers, stroke unit staff, wider hospital staff and community volunteers met on 4 to 6 occasions to plan and oversee development and implementation of improvements to increase social, cognitive and physical activity opportunties. In each site data were generated by two researchers pre and post-implementation of EBCD/AEBCD cycles through ethnographic non-participant observations of patient and staff activity at different times of the day and evening and at weekends; semi-structured audio or video interviews were conducted with a purposive sample of stroke unit staff, stroke survivors who had been inpatients in the units in the six months prior to EBCD/AEBCD cycles and their carers. Interviews were repeated post EBCD/AEBCD implementation. Patient reported outcome (PROM) and patient reported experience measures (PREM) were sent to 60 consecutive patients cared for in each unit in the six months pre and post implementation the AEBCD cycle. Behavioural Mapping was utilised to sample social, cognitive or physical activity levels for small groups of inpatients in each site pre and post implementation of EBCD/AEBCD.

Data analysis

Qualitative data were managed in NVivo 10[39] and analysed thematically[40]. Themes were identified and reviewed at each site and then discussed by the core research team (FJ, DJC,KWG,SH) in face-to-face meetings every 3 months. Summary memos for data sets in each site provided the basis for within and between site comparisons pre and post EBCD/AEBCD cycles. These were reviewed with the core

For peer review only - http://bmjopen.bmj.com/site/about/guidelines.xhtml

and wider research team, then discussed with SSC members. The qualitative data contributed to the process evaluation, see below. Quantitative data were managed using descriptive statisitics[35].

Ethical approval was provided by the Health Research Authority and South East Coast Brighton & Sussex Research Ethics Committee (16/LO/0212). Informed consent was obtained for all research activities.

Contexts

Stroke units were purposively selected, sampling focused on units outside large teaching hospitals, not already taking part in large clinical trials, that could commit to participation in a study comprising multiple stages over 6-9 months. Sites were stroke units receiving patients after care in a hyper-acute unit in the same hospital (sites 2 and 4) or a major stoke centre (sites 1 and 3) see Table 1. Biannual national Acute Stroke Organisational audit data[41] indicated the units performed within the mid-range across key quality indicators and were subject to the same staffing pressures and increasing caseload complexity reported nationally. Clinical leads at each site acted as principal investigators or supported the study.

Insert Table 1: Unit characteristics about here

Participants

130 staff, 53 stroke survivors and 26 carers participated in the CREATE study (Table 2)[35]. 366 hours of ethnographic observations were completed. Interviews were conducted with 76 staff, 53 stroke survivors and 27 family members/carers pre or post EBCD/AEBCD implementation. 43 co-design meetings were held across sites involving 23 stroke survivors, 21 family carers and 54 staff including physiotherapists, occupational therapists, speech and language therapists, dieticians, nurses, rehabilitation and support workers, hospital managers, support staff and volunteers. Behavioural mapping was completed with 68 inpatients resulting in 6,031 observations, and 181 stroke survivors returned PROM/PREM questionnaires.

Insert Table 2: Participants about here

Process Evaluation

In the CREATE study, preparation for and introduction of EBCD/AEBCD into the stroke units was an integral part of developing and implementing changes designed to increase activity opportunities. Normalisation Process Theory (NPT)[42-43] underpinned the process evaluation which encompassed the use of EBCD/AEBCD. NPT is concerned with understanding how complex interventions are

For peer review only - http://bmjopen.bmj.com/site/about/guidelines.xhtml

implemented and integrated into existing healthcare systems and is conceptualised through four generative mechanisms each with four components (Table 3). These mechanisms represent what participants 'do' to get the work of implementation done. They can be understood as a process (not necessarily linear) in which participants make sense of a new or different way of working, commit to it, make the effort required to work in that way and undertake continuous evaluation. NPT was used in two ways, firstly to guide data generation at each site (Supplementary file 1) and secondly as a sensitising lens in ongoing data analysis. NPT's constructs were used to identify and reflect upon processes that may act as facilitators or barriers to using EBCD/AEBCD to develop and implement interventions to increase activity opportunities for inpatient stroke survivors.

Insert Table 3 *Normalisation Process Theory about here*

Process evaluation data sources

A data generation plan linked to NPT's constructs identified data to be generated pre and post EBCD/AEBCD processes in each site, including participants' engagement in and experience of each element of EBCD/AEBCD cycles (Supplementary file 1). Researchers' observations of staff training in EBCD/AEBCD, researchers' reflections on facilitating EBCD/AEBCD cycles, and researchers' informal and formal engagement with participants in sites as part of recruitment activity and in generating data through observations and interviews were included. Demographic data for participants can be found in Supplementary file 2.

Process evaluation data analysis

Analysis comprised on-going integrative analysis by the same core team of researchers (FJ, KGW, DJC, SH) using NVivo10[37] as described above for the wider CREATE study, followed by review of the full data set. Analysis was underpinned by the use of NPT's constructs and subcomponents in order to examine the 'work' staff, former stroke survivors, carers and researchers were involved in to develop and implement increased activity opportunities. Once EBCD/AEBCD activities ceased in all sites, summary memos with researcher reflections were used to construct a single integrated account. Confirmability occurred through independent, then joint and team half-day analysis cycles, followed by discussing emerging findings with SSC members. Credibility and transferability are evident in the use of data extracts to support explanations of observational and interview data in terms of participants' engagement with EBCD/AEBCD, as well as the facilitators and barriers to developing and implementing increased activity opportunities in the four sites.

For peer review only - http://bmjopen.bmj.com/site/about/guidelines.xhtml

Process evaluation findings

Data generated through non-participant observations, semi-structured interviews and written feedback from EBCD/AEBCD events indicated positive experiences of participation in the EBCD/AEBCD processes. Most participants perceived that engaging in EBCD/AEBCD processes had led to changes which were increasing opportunities for independent and supervised activity in all sites. These changes affected not only inpatient stroke survivors' experiences but also those of their family members/carers and stroke unit staff. We now turn to an evaluation of the processes that underpinned the reported and observed changes. NPT mechanisms rarely operate in a linear or standalone way. In table 4 and the text below we highlight the various combinations of mechanisms we identified occurring within and across sites during the EBCD/AEBCD process and which in turn were identified as directly and indirectly helping to increase activity opportunities.

Insert table 4 about here:

Coherence and cognitive participation: making sense of and engaging with EBCD/AEBCD over time

Prior to research activity, members of each stroke team attended EBCD/AEBCD training led by the Point of Care Foundation[28]. These staff, supported by a researcher, were to lead EBCD/AEBCD implementation at their site. Full day training for sites 1 and 2 introduced the CREATE study and the theory and practice of EBCD/AEBCD; sites 3 and 4 received half-day training focused on implementation. Participants in the latter heard first-hand of how sites 1 and 2 were working to increase activity and discussed changes made by these teams.

> *"it was helpful meeting other people involved in the study on their wards, what they did and how it involved patients" (AEBCD training participant feedback site 3).*

Training afforded these staff the opportunity to start making sense of EBCD/AEBCD and to think individually and collectively about how to share their knowledge and understanding with colleagues, about what activity opportuntities were possible and what these would entail practically for the day-to-day work of their colleagues and themselves. Training enabled reflection on work with patients and carers using EBCD/AEBCD and how it differed from written feedback about patients' and carers' experiences. However, ensuring other stroke unit staff understood and engaged with the EBCD/AEBCD process was more challenging. There was no requirement for cascade training or other forms of knowledge sharing. In addition, changes to United Kingdom Health Research Authority (HRA) and Research Ethics Committee (REC) approvals processes led to a delay of four months between training site 1 and 2 staff and commencing EBCD activity, early interest quickly dissipated; understanding of what the EBCD approach would mean for wider staff groups' day-to-day work was limited.

For peer review only - http://bmjopen.bmj.com/site/about/guidelines.xhtml

However, researcher presence during pre-EBCD/AEBCD observation and interview periods enabled wider groups of staff to engage with and make sense of the project. Staff were curious about data generation methods used including behavioural mapping and observations, researchers used these opportunities to explain EBCD/AEBCD. Patients and carers also became aware of the study through the conduct of observations and behavioural mapping; they sought study information and expressed views on activity outside of therapy.

Coherence, cognitive participation and collective action: the faciliated EBCD/AEBCD process

In sites 1 and 2 the structured and facilitated approach of EBCD was a major factor contributing to staff progressing to a more engaged position, readier to commit to considering how change could happen in their site and to thinking through who needed to be involved. Separate and then joint- meetings enabled large numbers of staff to participate in EBCD events (Figure 1). They viewed trigger films, heard first-hand accounts of patients' and carers' experiences, worked in small groups with colleagues, former patients and carers to identify 'touch points' and agreed priorities for action. These were high-energy meetings with shared enthusiasm for change, and proved a powerful catalyst for larger groups of staff to share the view that change to stroke unit environments, access to resources, and routine-working practices to increase patient activity was possible. These events led to understanding of the role of co-design groups as being to work on ideas for activity opportunities generated by participants in joint meetings. In sites 3 and 4, starting the AEBCD process at the later joint-meeting stage meant knowledge and understanding was more limited initially. However, the joint-meeting still proved important in engaging staff in the process, particularly as information on how sites 1 and 2 had addressed issues identified in trigger films were shared at these meetings.

> *"It felt quite exciting - it will be interesting to see how it develops, keen to be involved and contribute" (Feedback after Joint event, staff, site 3)*

Other features of EBCD/AEBCD that facilitated cognitive participation and collective action, mainly in co-design group members, were the defined and time limited nature of the EBCD/AEBCD process. Clinical leads and service managers understood EBCD/AEBCD was a tried and tested service improvement model, this legitimised staff time and resource allocation committed to EBCD/AEBCD and associated improvements. Staff in all sites noted EBCD/AEBCD contrasted with previous attempts to introduce change, which were often 'poorly defined' in terms of timescales, roles and responsibilities and operated without additional resources. EBCD/AEBCD's participatory approach appeared to add a sense of responsibility for staff to deliver on agreed actions, and not to 'let down' patients and carers they worked with in co-design groups. As commitment to increasing activity opportunities grew, small

For peer review only - http://bmjopen.bmj.com/site/about/guidelines.xhtml

groups of staff, not all of whom were stroke unit based, worked together to progress actions agreed by co-design groups.

Cognitive participation and collective action: patient and family member participation in EBCD/AEBCD

EBCD/AEBCD ensures patients and carers express their priorities for change and engages them as equal partners in designing and implementing solutions. In sites 1 and 2 separate patient and carer meetings provided opportunities to explore experiences of stroke and activity/inactivity after stroke. Trigger films demonstrated commonality and difference in experiences and began the process of identification by these former patients and carers as a group, with a shared belief in changing patients' activity experiences in their local stroke units. Although, former patients and carers in sites 3 and 4 did not have local trigger film participation in common they recognised the long periods of inactivity described in the site 1 and 2 trigger films and lack of activity opportunities as similar to their own; trigger films validated important issues raised. EBCD/AEBCD provided former patients and carers with the means and confidence to give voice to their experiences and appeared to help these participants understand how they could work with staff as partners in bringing about improvements.

We felt able to say what we wanted to say and what we wanted to say has turned out to have a valuable effect, so, yeah, very happy to say that we didn't feel intimidated in any way (Carer-Site 4, post).

Participants in EBCD/AEBCD shared a common experience with their peers and, with the staff with whom they exchanged ideas and problem solved in co-design groups. This also appeared true for managers, professional leads and volunteers who were 'external' to the day-to-day work of stroke units but involved in EBCD/AEBCD events. Patients' voices and the authentic examples depicted in trigger films were critical in all staff reorientating their focus to patients' experiences. These events contributed to participants' progression from shared understanding of the importance of change to working together to define ways to implement ideas into existing working practices. This included thinking through how changes to increase activity would be actioned, who would take responsibility and, who would routinely deliver these actions. These mechanisms represent progression through cognitive participation and collective action over time. Clinical staff facilitated all co-design groups, patients or carers did not seek to lead; their contributions were actively pursued, and observational and interview data findings confirm these were influential in improvements that occurred. Examples included designing and painting murals (sites 1 and 3) and the redesign of patient and carer facing documentation (sites 2 and 4), actions designed to facilitate increased activity through providing

For peer review only - http://bmjopen.bmj.com/site/about/guidelines.xhtml

stimulating spaces and provide information about the purpose of new spaces and opportunities to undertake activity.

Cognitive participation and collective action: wider staff groups have less involvement in planning and actioning change

Co-design groups brought together those who believed in and were committed to making change happen. These small, mainly self-selected groups provided a stimulus and workspace for staff, former patients, family members/carers and volunteers to engage with and commit to planning and actioning changes to increase activity opportunities and drive new working practices. However, in the early months of the study it was difficult for staff not involved in co-design groups to envisage whether or how increasing patient activity may affect their roles and working practice. Coherence and cognitive participation, linked to commitment to change and comprehension of the possible benefits of changes in individual and collective practice, developed at different rates across wider staff groups at all sites.

Early 'wins' such as a new 'Social Corner' at site 1 raised awareness amongst all staff, inpatients and visiting family/carers that change was happening. In terms of collective action, some staff incorporated the new space in therapeutic activity or prompted its use independently or with family. Similarly, changing a room at site 4 from a wheelchair storage area to a day-room was visible evidence of change and led to its routine use for independent and supervised activity. Such 'public' examples enabled wider understanding of how EBCD/AEBCD led to implementation of environmental and practice changes; this stimulated additional discussion among some staff groups about ways to incorporate increasing activity into day-to-day practice:

There's been staff involved from physiotherapy and occupational therapy, we meet in a morning for a board meeting and we've handed over what progressions have been from the CREATE study. So say for example we're telling them about the updating of the garden and if we need to take anybody to the garden (Co-design group member, site 4 post).

Communication and engagement across nursing teams working early, late, on weekends, and night duty was more difficult to achieve; observations indicated project information and changes made were not routinely included in nursing handovers at any site; this initially limited coherence and cognitive participation among nursing staff.

Collective action: challenges in implementing co-designed improvements

One challenge of EBCD/AEBCD is the relatively long time period over which change is planned, implemented and evaluated. Managers largely encouraged staff to participate in EBCD/AEBCD but did

For peer review only - http://bmjopen.bmj.com/site/about/guidelines.xhtml

not allocate time for participation; staff were encouraged to 'work flexibly'. For nursing staff and HCSWs, attending co-design meetings during shifts was problematic. In site 1 staff sickness and workload challenges meant that three of four EBCD trained staff did not participate in co-design work. At site 4 two AEBCD trained staff, both nurses, attended the joint-meeting but did not participate directly in co-design groups thereafter. Staff led co-design groups in all sites experienced similar challenges but other staff volunteered to take responsibility. Enthusiasm for the project across all sites meant some staff attended joint-meetings on their days off and in sites 1-3, some staff participated in co-design meetings on days off or before shifts. Most staff in co-design groups reported completing activities in their own time. Where implementation did not require substantial change in roles and was perceived as enhancing patients' experience consistent with rehabilitation, staff indicated the extra effort was worthwhile.

"I think it's given me a massive workload, I think it's doubled it...to be fair. But, I was committed, I mean I took it on, but I've enjoyed that, I'm glad for the changes. [.....]I knew we needed changes, so I was happy to help bring the changes". (Staff, site 4, post).

As changes became visible to wider staff groups and involved patients more regularly, staff appeared more receptive to involving external partners. Examples include complementary therapy from a local health network (site 1), fortnightly singing with local University students (site 2) and musicians from a community arts group (site 3). Changes elicited positive and negative responses. At site 2 some staff complained the 'Something About Me' (patient information) board mounted behind patients' beds appeared without consultation. However, these staff also said they liked what the board was designed to do. Comments like this highlight how difficult it is to secure cognitive participation and commitment to collective action in staff who have limited engagement with implementation of co-designed interventions. Despite some of the challenges outlined, as activity opportunities became more evident shifts in staff perceptions and behaviours suggested collective action and reflexive monitoring were becoming established in most units. Overall team members' perceived changes and acknowledged the positive impact on patients' independence, completion of personal care tasks or engagement in therapy.

Cognitive participation and collective action: influence of managerial authority

Managerial support for service improvements generated through EBCD/AEBCD[36] is important. In sites 1 and 2 researchers set up oversight groups including senior managers, matrons/senior nurses and staff with cross-organisational roles. The commitments of these managers meant interaction was

For peer review only - http://bmjopen.bmj.com/site/about/guidelines.xhtml

largely through email updates or one-to-one meetings. Oversight groups could not be established at sites 3 or 4 despite invitations to attend AEBCD activity. Oversight group members in sites 1 and 2 supported project activity, helped navigate complex NHS Trust organisational structures and, in specific situations, provided resources to enable co-design group ideas to progress. Having defined senior managerial contacts ensured unit-based staff could activate these lines of support and translate ideas into collective action. This was more difficult in sites 3 and 4 where only the Chief Executive and a therapy lead engaged with AEBCD groups. Site 3 staff completed a sponsored run and the Chief Executive matched the sum raised. Funds (£8000) were used to redesign a day room previously only used by staff and partly to install a kitchenette for patient and family member/carer use.

In all sites, staff working outside of stroke units, including therapy service managers, matrons, patient experience managers, patient safety officers, volunteer co-ordinator and estates managers joined co-design groups. Although, not involved in every meeting their participation was often significant in terms of cognitive participation and collective action. NHS staff are acutely aware of resource constraints affecting their services, and most have experienced frustration at organisational barriers to improving services. Barriers identified included infection control and patient safety requirements, delays or inaction when estates work is requested and bureaucratic processes associated with including volunteers in unit-based activity. In early co-design meetings at all sites, staff that were otherwise dynamic and enthusiastic advocates for increasing patient activity often expressed the view that such barriers were fixed and would limit what could be achieved. The perception that 'the NHS Trust would not 'allow', for example, painting murals, adding shelving in patient bays, adding hot drinks facilities for patients and carers to use independently or having volunteers to support patients with social eating, was pervasive. In most sites these perceptions proved to be largely inaccurate; staff from the wider hospital, patient services and volunteer services typically explained how changes could in fact be realised, and importantly, provided examples of where such changes were already operating in the same hospital.

Coherence, cognitive participant and collective action: Facilitating EBCD/AEBCD:

Researchers organised staff, patient and joint events in consultation with EBCD/AEBCD trained staff, recruited former stroke patients and carers and ensured they could attend co-design groups. They booked accessible meeting rooms, arranged reimbursement for patients' and carers' expenses or arranged transport to and from meetings; without this level of support key EBCD/AEBCD events may not have occurred. Researchers co-facilitated staff and joint-meetings; although often initiated by researchers, the joint approach built confidence in EBCD/AEBCD trained staff. In sites 1 and 2 this

For peer review only - http://bmjopen.bmj.com/site/about/guidelines.xhtml

increased engagement of key staff in EBCD activities. For sites 3 and 4, AEBCD trained staff had less opportunity to work with researchers prior to joint-meetings. In site 3 this appeared to have little impact on co-design meetings, whereas in site 4 team members were less confident in leading co-design meetings. At all sites newsletters were produced by researchers or core team member to share work of co-design groups to wider staff and patient groups (Supplementary file 3).

Collective action and reflexive monitoring: implementing and embedding co-designed changes into stroke unit practice

It took 3-4 co-design groups for staff to recognise potential for changes in spaces for activity and to facilitate supervised and independent activity. For most therapists and TAs increasing activity was conceptualised not only as reducing boredom and occupying patients' time but as a therapeutic opportunity. Social activity focused on lunch and breakfast groups at sites 2 and 4, provided opportunities to work on therapy goals including cognitive challenge and functional task practice. These were consistent with therapists' aims in rehabilitation, did not require working in new or different ways and were embraced. In some sites staff reported changes such as the 'Something About Me board' (site 2) or the 'home in the ward' personalisation of bed spaces (sites 1 and 3) made therapy more relevant as staff could draw on information made available to them through these methods. Over time, the work of implementation became more focused on embedding regularly occurring group or individually focused activity into therapists and TAs work although the rate at which this occurred varied across sites.

However, post EBCD/AEBCD observations and interviews suggested limited interdisciplinary consideration of activity promotion. Observational and interview data indicated that initially many nurses did not develop an individual or collective understanding of EBCD/AEBCD as something they needed to participate in. This suggested that some nurses team members did not engage with the view that increasing participation in social, cognitive and physical activity was consistent with their view of what constitutes nursing work in stroke units. However, nursing staff overall expressed support for the intentions of the co-design groups but the challenges involved in nurses (in all sites) being able to attend these groups are likely to have impacted on the nurses' collective understanding and engagement in the EBCD/AEBCD process. Ward managers were aware of the challenges Registered nurses (RNs) faced in taking time out of nursing care provision during 8 or 12 hour shifts allocated time for RNs to attend and participate in co-design meetings. RN and HCSW attendance occurred at site 2 but not in other sites. Observations highlighted nursing staff in each site had high workloads and were frequently affected by staff shortages. However, in all sites there were periods in

For peer review only - http://bmjopen.bmj.com/site/about/guidelines.xhtml

each day typically late morning and late afternoon when many RNs and HCSWs congregated at shared workstations. Organising co-design meetings and EBCD/AEBCD updates at these times when nursing staff appeared less busy may have facilitated greater involvement and engagement. Overall, there was limited evidence of RN's encouraging patient focused social or cognitive activity. There were exceptions, two RNs and a HCSW at site 2 actively engaged in co-design work and, toward the end of AEBCD activity, nurses at site 4 routinely worked with therapists in daily breakfast groups. It is possible that as therapist and family led activity increases in these units greater interdisciplinary participation will follow.

Discussion

The CREATE study process evaluation findings confirmed EBCD/AEBCD[26,28,33-34] was feasible to use in stroke units and that this approach facilitated development and implementation of increased activity opportunities. There were four main factors influencing engagement in EBCD/ABCD. Firstly, the participatory approach ensured groups of staff in each unit worked directly with former patients and family/carers over a sustained period to jointly address issues key to increasing social, cognitive and physical activity levels post-stroke. Secondly, the structured, facilitated and time limited nature of EBCD/AEBCD enabled each service to prioritise and agree locally appropriate changes. Thirdly, at the outset of the study photographic evidence gathered by researchers highlighted the typically cold, dark and clinical appearance of the stroke units. Linked to this, participants in all sites chose to work on changing physical environments before focusing on specific types of post-stroke activity. Lastly, EBCD/AEBCD made patient and family/carer experiences real in ways brief questionnaires cannot. Trigger films[26,28] confronted staff, often for the first time in their careers, with the fears, frustrations, and positive and negative reality of stroke survivors and family/carers. This proved catalytic in securing staff commitment to bring about change. In all units, those directly involved in EBCD/AEBCD were positive about the experience and the perceived changes underway in the units. These findings are consistent with those identified in a rapid review of outcomes of co-production studies in acute healthcare settings[36]. The shared sense of purpose and experience developed in co-design groups was consistent with what is termed a 'community of practice'[44] and led to development and implementation of innovative solutions to problems identified by patients and family/carers. Staff more peripheral to EBCD/AEBCD reported some lack of understanding of and scepticism about the process early in the study. However, towards the end of EBCD/AEBCD processes, in interview and during observations most staff indicated positive views on the implementation and embedding of co-designed environmental and practice changes in sites.

For peer review only - http://bmjopen.bmj.com/site/about/guidelines.xhtml

Drawing on NPT's mechanisms as a sensitising device in collection and analysis[42-43] of our data focused attention on individual, group and organisational factors facilitating the use of EBCD/AEBCD to develop and support the implementation and potentially the longer-term sustainability of changes introduced. These included engagement of EBCD/AEBCD trained staff with the skills, expertise and resources of key staff from the wider hospital, including patient experience managers, volunteer coordinators and estates managers. Providing regular project information and updates for managers throughout the EBCD/AEBCD process was instrumental in maintaining their support. Both activities were instrumental in stroke unit staff progressing from understanding the potential of co-producing change with stroke survivors and family/carers (coherence and cognitive participation) through committing personally and as staff groups to the kinds of shifts in work patterns needed to facilitate increased patient activity opportunities. These activities together with managerial support were linked to stimulating and supporting collective action in sites.

An additional facilitator supporting implementation of improvements in these sites was engagement with local communities. This occurred at different times and at different levels across the units; sometimes driven by family members, sometimes by patient experience managers or volunteer co-ordinators with links to colleges, charities or voluntary organisation. In each unit, there were tangible benefits from community engagement and community groups welcomed the opportunity to provide support to their local hospital. The early evidence suggested this engagement supported collective action and reflexive monitoring with positive feedback from inpatients and family members helping sustain staff groups' commitment to embedding improvements in routine stroke unit practice.

Our findings concur with previous EBCD/AEBCD studies in highlighting the central role of site-based facilitators in establishing and maintaining the EBCD/AEBCD process which in this study were integral to developing and implementing increased activity opportunities[34,36,45-46]. Different models are reported but in the CREATE study researchers undertook this role at each site; they spent less time in sites than reported in other studies, and undertook facilitation alongside data collection. Given the high workload demands and clinical priorities for all stroke unit staff in the study sites, it is unlikely that the EBCD/AEBCD process would have progressed without researcher facilitation. Drawing on NPT highlighted that implementation, in this case of both the EBCD/AEBCD process and the implementation of improvements resulting from the process was not a simple linear process and progressed at different rates in each site.

Barriers to EBCD/AEBCD implementation in all sites were broadly similar to those reported in relation to implementing complex interventions in health services[47-49]. These included staff and local organisational changes at three of the four sites. Less than optimum staffing levels combined with the high level of dependency of stroke survivors in these units made it challenging to free staff to

For peer review only - http://bmjopen.bmj.com/site/about/guidelines.xhtml

participate in larger EBCD/AEBCD events including joint meetings and celebratory events. Similarly, although fewer staff were required to participate in co-design meetings the frequency, duration and additional workload generated by these meetings proved challenging for EBCD/AEBCD trained and other staff to integrate into their working day. These factors, impacted in particular in NPT terms, on coherence and cognitive participation; understanding the process and potential of the participatory change approach and commiting to that at a personal and practice level often developed slowly in staff not directly involved in co-desing groups. In addition, EBCD or AEBCD approaches were underway for nine and six months respectively, and engaging all stroke unit staff in developing and implementing improvements proved difficult at times. Nonetheless, staff did not resist or sabotage the EBCD/AEBCD process or subsequent changes. The key factor in increasing cognitive participation and progression to collective action was the increasing visibility of changes agreed in co-design meetings. As activity opportunities were integrated into staffs daily or weekly routines, independent and family/carer or volunteer supported patient activity also increased in newly created spaces on the stroke units. At the end of the study these changes appeared to be impacting on unit cultures and staff practice, in particular more group activities were occurring and were increasingly part of the thinking of staff, particularly therapists, but also with some nursing involvement in each unit.

This process evaluation differed from some other complex intervention evaluations in two ways. Firstly, the use of logic models to define an intervention, its anticipated mechanisms of action and to frame research questions and methods in process evaluation is commonly advocated [38]. However, given the defined approach, stages and structured activities and mechanisms of action identified in previous EBCD/AEBCD studies, a logic model was not developed in the CREATE process evaluation. Secondly, the researchers conducting the process evaluation were members of the core research team rather than working independently of that team. This ensured that researchers were able to both participate in and observe EBCD/AEBCD activities. However, we acknowledge that in working closely with staff, former patients and family/carers, researchers were themselves part of the EBCD/AEBCD process and the design and implementation of changes they were evaluating.

Conclusion

The findings from the CREATE study, the first of its kind in stroke services, suggest using a co-production approach was instrumental in creating conditions for locally determined former patient and family/carer led change and innovation in service provision. It was possible to utilise EBCD/AEBCD

For peer review only - http://bmjopen.bmj.com/site/about/guidelines.xhtml
For peer review only

in stroke units providing post-acute and rehabilitation care; this facilitated development and implementation of environmental changes and revisions to work routines which supported increased activity opportunities. NPT's mechanisms were instrumental in identifying facilitators and barriers at the individual, group and organisational levels and attending to these will benefit future implementations of similar approaches. The introduction of EBCD/AEBCD as part of a funded research programme legitimised and supported the co-production activity in these units. However, with appropriate facilitation, managerial engagement and use of wider hospital resources, the approach could be utilised in other rehabilitation stroke services and similar post-acute inpatient environments.

Acknowledgements:
The authors acknowledge the contribution of the stroke survivors, family members, stroke unit staff, volunteers, hospital managers and local community groups and the support for the study of the four participating NHS Foundation Trust hospitals.

We also wish to acknowledge colleagues who supported elements of the CREATE study, these include Dr Tino Kulnik who conducted pre-implementation interviews for site 1. Dr Alessia Costa, supported ethnographic fieldwork in site 1. Carole Pound, supported co-design events in site 1.

Funding statement: This study was funded by the National Institute for Health Research (NIHR) [under its Health Services and Delivery Research (HS&DR) Programme. Grant Reference Number: 13-11/495]. The views expressed are those of the author(s) and not necessarily those of the NIHR or the Department of Health and Social Care.

Competing interests statement: GR teaches on the EBCD training courses run by 'The Point of Care Foundation' in London. GR is one of the originators of EBCD. The remaining authors declare no other competing interests.

Data sharing statement: No additional data are available.

Author contributions: The CREATE study and embedded process evaluation were designed by FJ, DJC, GR, CMc, RH, GCC and AMc. FJ, DJC, SH and KGW collected and analysed the data. AMc, CMc and RH participated in some joint meetings and celebration events as part of EBCD/AEBCD cycles. DJC developed the manuscript, FJ, GR, CMc, AMc, RH, GCC, SH and KGW reviewed and edited the manuscript. All authors read and approved the final manuscript.

For peer review only

References

1. Feigin VL, Forouzanfar MH, Krishnamurthi R, et al, on behalf of the Global Burden of Diseases, Injuries, and Risk Factors Study 2010 (GBD 2010) andthe GBD Stroke Experts Group. Global and regional burden of stroke during1990–2010: findings from the Global Burden of Diseases Study 2010. Lancet. 2014; 383(9913):245-255.
2. Stroke Association, UK. State of the Nation. Stroke Statistics. Online. Available at: https://www.stroke.org.uk/sites/default/files/state_of_the_nation_2018.pdf Accessed 10.02.2020
3. Stroke Unit Trialists' Collaboration. Organised inpatient (stroke unit) care for stroke. Cochrane Database of Systematic Reviews 2013, Issue 9. Art. No.: CD000197. DOI: 10.1002/14651858.CD000197.pub3.
4. Keith RA. Activity patterns of stroke rehabilitation unit. Soc Sci Med.1980; 14:575-580.
5. Keith, R.A. Cowell,K.S., Time use of stroke patinets in three rehabilitation hospitals. Social Science and Medicine. 1987. 24,6:529-533.
6. Lincoln NB, Gamlen R, Thomason H. Behavioural mapping of patients on a stroke unit. Int Disabil Stud.1989; 11:149-154.
7. Bernhardt J, Dewey H, Thrift A & Donnan G. Inactive and alone: physical activity within the first 14 days of acute stroke unit care. Stroke 2004:35;1005-9.
8. Janssen H, Ada L, Bernhardt J, McElduff P, Pollack M, Nilsson M, Spratt NJ (2014). An enriched environment increases activity in stroke patients undergoing rehabilitation in a mixed rehabilitation unit: a pilot non-randomized controlled trial. Disabil Rehabil. 36: 255-262.
9. Prakash, V., Shah, M. A., and Hariohm, K. (2016). Family's presence associated with increased physical activity in patients with acute stroke: an observational study. *Braz. J. Phys. Ther.* 20, 306–311. doi: 10.1590/bjpt-rbf.2014.0172
10. Rosbergen, I. C. M., Grimley, R. S., Hayward, K. S., Walker, K. C., Rowley, D., Campbell, A. M., et al. (2017). Embedding an enriched environment in an acute stroke unit increases activity in people with stroke: a controlled before-after pilot study. *Clin. Rehabil.* 31, 1516–1528. doi: 10.1177/0269215517705181
11. Foley N, McClure JA, Meyer M, Salter K, Bureau Y, Teasell R. Inpatient rehabilitation following stroke: amount of therapy received and associations with functional recovery. Disabil Rehabil 2012; 34(25): 2132-8.

For peer review only - http://bmjopen.bmj.com/site/about/guidelines.xhtml

12. Otterman NM, van der Wees PJ, Bernhardt J, Kwakkel G. Physical therapists' guideline adherence on early mobilization and intensity of practice at Dutch acute stroke units a country-wide survey. Stroke 2012; 43(9): 2395-401.
13. Merchant, J., Kitsos,G., Ashby,S., Kitsos,A., Hubbard, I. Occupational therapy and physiotherapy in acute stroke: do rural patients receive less therapy? Stroke Research and Treatment. 2016. http:/dx.doi.org/10.1155/2016/1582706.
14. Clarke,D,J.; Burton,L.J.; Tyson,S., Rodgers,H., Drummond,A., Palmer,., Hoffman,A., PrescottM., Tyrrell, P., Brkic, L., Grenfell,K., Forster,A. Why do stroke survivors not receive recommended amounts of active therapy? Findings from the ReAcT study, a mixed-methods case-study evaluation in eight stroke units. Clinical Rehabilitation 1–14. 2018 DOI: 10.1177/0269215518765329
15. Fini NA, Holland AE, Keating J, Simek J, Bernhardt,J. How Physically Active Are People Following Stroke? Systematic Review and Quantitative Synthesis. Phys Ther. 2017 Jul 1; 97(7):707-717.
16. Luker J, Murray C, Lynch E, Bernhardsson S, Shannon M, Bernhardt J. Carers' Experiences, Needs, and Preferences During Inpatient Stroke Rehabilitation: A Systematic Review of Qualitative Studies. Arch Phys Med Rehabil. 2017;98(9):1852–62 e1813.
17. Ellul, J., Watkins, C., Ferguson, N., Barer, D., and Rowe,J. "Increasing patient engagement in rehabilitation activities". Clinical Rehabilitation. 1993. 7(4):297-302.
18. Newall, J.T., Wood, V.A., Hewer. R,L., and Tinson, D,J. (1997). "Development of a neurological rehabilitation environment: an observational study". Clinical Rehabilitation. 11(2): 146-55.
19. Rosbergen, I. C., Grimley, R. S., Hayward, K. S., Walker, K. C., Rowley, D., Campbell, A. M., McGufficke, S., Robertson, S. T., Trinder, J., Janssen, H., & Brauer, S. G. (2016). The effect of an enriched environment on activity levels in people with stroke in an acute stroke unit: protocol for a before-after pilot study. *Pilot and feasibility studies*, *2*, 36. https://doi.org/10.1186/s40814-016-0081-z
20. Khan,F. Amatya,B., Elmalik,A, Lowe,M., Ng,L., Reid,I., Galea,M.P. et al, An Enriched Environmental Programme During Inpatient Neuro-rehabilitation: A Randomized Controlled Trial. Journal of Rehabilitation Medicine, 48, (5): 417-425(9)2016;
21. Rosbergen, I. C., Grimley, R. S., Hayward, K. S., & Brauer, S. G. (2019). The impact of environmental enrichment in an acute stroke unit on how and when patients undertake activities. Clinical Rehabilitation, 33(4), 784–795. https://doi.org/10.1177/0269215518820087
22. McDonald, M. W., Hayward, K. S., Rosbergen, I., Jeffers, M. S., & Corbett, D. (2018). Is Environmental Enrichment Ready for Clinical Application in Human Post-stroke Rehabilitation?. Frontiers in behavioral neuroscience, 12, 135. doi:10.3389/fnbeh.2018.00135.
23. Anåker, A., von Koch, L., Sjöstrand, C., Bernhardt, J., & Elf, M. (2017). A comparative study of patients' activities and interactions in a stroke unit before and after reconstruction-The significance of the built environment. PloS one, 12(7), e0177477. https://doi.org/10.1371/journal.pone.0177477
24. Shannon,M.M., Elf,M., Churilov,L., Olver,J., Pert,A., Bernhardt,J. Can the physical environment itself influence neurological patient activity?, Disability and Rehabilitation, 2019. 41:10, 1177-1189, DOI: 10.1080/09638288.2017.1423520
25. Needham, C. and Carr, S. Co-production: an emerging evidence base for adult social care transformation. Research Briefing 31. 2009. Social Care Institute for Excellence.
26. Robert G , Cornwell J , Locock L , et al . Patients and staff as co-designers of healthcare services. BMJ 2015;350:g7714.
27. Batalden P. Getting more health from healthcare: quality improvement must acknowledge patient coproduction—an essay by Paul Batalden. BMJ. 2018 Sep 6;362:k3617.
28. Point of Care Foundation, Experience-based co-design toolkit, Available from:https://www.pointofcarefoundation.org.uk/resource/experience-based-co-design-ebcd-toolkit/ Accessed 10/02/2020.
29. Coldham,T., Denegri, S., Green,G. Staniszewska,S., Tembo,D., Torok,K. and Turner,K. 2018. Guidance on co-producing a research project. Southampton, INVOLVE. https://www.invo.org.uk/posttypepublication/guidance-on-co-producing-a-research-project/ Accessed 10/02/2020

For peer review only - http://bmjopen.bmj.com/site/about/guidelines.xhtml

30. Boaden, R. Push, pull or co-produce? Journal of Health Services Research and Policy.2020. 25 (2): 67-69
31. Co-production of health and wellbeing Scotland. Online: http://www.coproductionscotland.org.uk/about/what-is-co-production/ Accessed 10/02/2020
32. Bate SP , Robert G . Bringing user experience to health care improvement: the concepts, methods and practices of experience-based design. Oxford: Radcliffe Publishing, 2007.
33. Donetto,S., Tsianakas,V, Robert,G. Using Experience-based Co-design (EBCD) to improve the quality of healthcare: mapping where we are now and establishing future directions. National Nursing Research Unit. Final Report. Kings College London. 2014.
34. Locock L, Robert G, Boaz A, Vougioukalou S, Shuldham C, Fielden J, *et al.* Testing accelerated experience-based co-design: a qualitative study of using a national archive of patient experience narrative interviews to promote rapid patient-centred service improvement. *Health Serv Deliv Res* 2014;2(4)
35.HS&DR 13/114/95: Using co-production to improve patient carer and staff experiences in health care organizations: a multi-centre, mixed methods evaluation in inpatient stroke units (CREATE). https://www.journalslibrary.nihr.ac.uk/programmes/hsdr/1311495/#/ .
36. Clarke D, Jones F, Harris R on behalf of the Collaborative Rehabilitation Environments in Acute Stroke (CREATE) team, et al What outcomes are associated with developing and implementing co-produced interventions in acute healthcare settings? A rapid evidence synthesis BMJ Open 2017;7:e014650. doi: 10.1136/bmjopen-2016-014650
37. Craig P, Dieppe P, Macintyre S, et al. Developing and evaluating complex interventions: new guidance: MRC, 2008.
38. Moore GF, Audrey S, Barker M, Bond L, Bonell C, Hardeman W, Moore L, O'Cathain A, Tinati T, Wight D, Baird J. Process evaluation of complex interventions: Medical Research Council guidance. BMJ 2015; 350: h1258.
39. NVivo 10 QSR International PTY 2015
40. Braun,V., Clarke,V. Using thematic analysis in psychology, Qualitative Research in Psychology,2006. 3:2, 77-101, DOI: 10.1191/1478088706qp063oa 2006
41. Royal College of Physicians. SSNAP Biannual national Acute Stroke Organisational audit data, reported in 2016. London, Royal College of Physicians.
42. May, C., Rapley, T., Mair, F.S., Treweek, S., Murray, E., Ballini, L., Macfarlane, A. Girling, M. and Finch, T.L. (2015) Normalization Process Theory On-line Users' Manual, Toolkit and NoMAD instrument. Available from http://www.normalizationprocess.org Accessed 10/02/2020.
43. May,C., Cummings, A., Girling,M., Bracher,M., Mair, F.S., May,C.M., Murray, E., Myall, M., Rapley, T., Finch, T. Using Normalisation Process Theory in feasibility studies and process evaluations of compelx healthcare interventions: a systematic review. Implementation Science. 2018. 13:80, DOI: 10.1186/s13012-018-0758-1
44. Wenger, E. 1998 Communities of Practice: learning, meaning, and identity. Cambridge University Press
45. Tollyfield, R. Facilitating an accelerated experience-based co-design project. *Brit Journal of Nurs*, 2014, 23, (3):136-141
46. Larkin, M Boden, ZVR, Newton, E et al, On the Brink of Genuinely Collaborative Care: Experience-Based Co-Design in Mental Health. *Qualitative Health Research* 2015, 25(11) 1463–1476

47. Grol R, Grimshaw J. From best evidence to best practice: effective implementation of change in patients' care. Lancet. 2003;362:1225–1230.

48. Grimshaw JM, Eccles MP, Lavis JNet al. . Knowledge translation of research findings. Implement Sci. 2012;7:50.
49. Child, S., Goodwin, V., Garside, R. et al. Implementation Sci (2012) 7: 91. https://doi.org/10.1186/1748-5908-7-91

For peer review only - http://bmjopen.bmj.com/site/about/guidelines.xhtml

For peer review only

For peer review only - http://bmjopen.bmj.com/site/about/guidelines.xhtml

For peer review only

For peer review only - http://bmjopen.bmj.com/site/about/guidelines.xhtml

Table 1 Unit characteristics

	Site 1 (EBCD)	Site 2 (EBCD)	Site 3 (AEBCD)	Site 4 (AEBCD)
**Number of stroke beds**	24	24	26	26 beds across two adjoining wards (14 and 12 beds)
**Hospital type**	District general hospital with 629 beds	District general hospital with 500 beds	District general hospital with 700 beds	District general hospital with 600 beds
**Number of stroke patients treated per year**	195	978*	250	640*
**Typical length of stay (days)**	28	13*	28	21*
**7-day therapy service**	No	An OT and PT worked on Saturday covering the acute and rehabilitation units. A Stroke Rehabilitation Assistant	No	No

For peer review only - http://bmjopen.bmj.com/site/about/guidelines.xhtml

		worked on the rehabilitation unit on Saturdays and Sundays.		
**Performance in National Acute Organisational Audit (RCP, 2017).**	Achieved 7 of the 10 key indicators.	Achieved 4 of 10 key indicators.	Achieved 8 of the 10 key indicators.	Achieved 5 of the 10 key indicators.

* Data for Sites 2 and 4 include data for Hyperacute/Acute units in the same hospital.

Table 2 Number of participants by data generation method

**Site**	**Staff interviews**	**Patient interviews**	**Carer interviews**	**Non-participant Observations (hours)**
**Site 1 pre EBCD**	N=13	N= 9	N= 4	50
**Site 1 post EBCD**	8	5	5	47
**Site 2 pre EBCD**	15	9	4	48

For peer review only - http://bmjopen.bmj.com/site/about/guidelines.xhtml

For peer review only

**Site 2 post EBCD**	7	6	2	44
**Site 3 pre AEBCD**	6	9	3	50
**Site 3 post AEBCD**	8	6	3	37
**Site 4 pre AEBCD**	7	4	3	44
**Site 4 post AEBCD**	12	5	3	46
**TOTAL**	**76**	**53**	**27**	**366 hours**

For peer review only - http://bmjopen.bmj.com/site/about/guidelines.xhtml

Table 3 **Normalisation Process Theory**

NPT constructs	Components	Explanation
**Coherence**	• Differentiation<br>• Communal specification<br>• Individual specification<br>• Internalisation	The sense making work that people do individually and collectively when faced with implementing changes to existing working practices. This would include differentiating new practices from existing work and thinking through not only the perceived value and benefits of desired/planned changes but also what work will be required of individual people in a setting to bring about these changes.
**Cognitive Participation**	• Initiation<br>• Enrolment<br>• Legitimation<br>• Activation	The work that people need to do to engage with and commit to a new set of working practices. This often requires bringing together those who believe in and are committed to making changes happen. This also involves people working together to define ways to implement and sustain the new working practices.
**Collective Action**	• Interactional workability<br>• Relational integration<br>• Skill set workability<br>• Contextual integration	The work that will be required of people to actually implement changes in practices, including preparation and/or training of staff. Often this entails rethinking how far existing work practices and the division of labour in a setting will have to be changed or adapted to implement the new practices. This requires consideration of not only who will do the work required, but also the skills and knowledge of people who will do the work and the availability of the resources they need to enact and sustain the new working practices.
**Reflexive monitoring**	• Systematisation<br>• Communal appraisal<br>• Individual appraisal<br>• Reconfiguration	Peoples' individual and collective on-going informal and formal appraisal of the usefulness or effectiveness of changes in working practices. This involves considering how the new practices affect the other work required of individuals and groups and whether the intended benefits of the new working practices are evident for the intended recipients and staff.

For peer review only

Table 4: NPT mechanisms and components identified during the EBCD/AEBCD process

Observed processes	Combinations of NPT mechanisms and components identified during the EBCD/AEBCD process	Facilitators influencing use of EBCD/AEBCD as a process to develop and implement improvements to increase activity opportunity	Barriers and challenges to using EBCD/AEBCD as a process to develop and implement improvements to increase activity opportunity
Staff, inpatients, former and carers made sense of and engaged with EBCD/AEBCD at different rates over time.	*Coherence:*<br>• *Differentiation*<br>• *Individual specification*<br><br>*Cognitive participation:*<br>• *Initiation*<br>• *Enrolment*	• Small groups of staff with access to EBCD/AEBCD team training;<br>• Researcher presence during observation and interview periods provided; opportunities to explain EBCD/AEBCD to more staff one to one and in small groups;<br>• Patients, former patients and carers became aware of the study through observations and interview participation.	• Without access to EBCD/AEBCD training -challenging in all sites to facilitate individual and collective understanding and engagement in wider stroke unit and hospital staff and volunteers;<br>• Delay between training and commencing EBCD/AEBCD activity in all sites- early interest in the study dissipated- impacted on understanding what EBCD/AEBCD process could achieve and would mean for wider staff groups;<br>• Inpatients did not participate in co-design meetings and had no direct in engagement creating increased activity opportunities.
The facilitated EBCD/AEBCD process was perceived to be different and more effective than prior change initiatives designed to improve activity opportunities.	*Coherence:*<br>• *Differentiation*<br>• *Individual specification*<br>• *Communal specification*<br><br>*Cognitive participation:*<br>• *Initiation*<br>• *Enrolment*<br>• *Legitimation*<br><br>*Collective action:*<br>• *Interactional workability*	• Structured, time limited and externally facilitated approach of EBCD/AEBCD;<br>• Sites 1 and 2: large numbers of staff participate in separate and joint meetings;<br>• Sites 3 and 4 (AEBCD) joint meetings important in engaging staff in the process;<br>• EBCD/ABCD as a tried and tested service improvement model- legitimised staff time and resource allocation.	• Across all sites limited numbers of stroke unit staff, wider hospital staff, volunteers, former patients and carers can participate in EBCD/AEBCD process activities.

For peer review only - http://bmjopen.bmj.com/site/about/guidelines.xhtml

Former patient & family member participation in EBCD/AEBCD	*Coherence:*<br>• *Individual specification*<br>• *Communal specification*<br><br>*Cognitive participation:*<br>• *Initiation*<br>• *Enrolment*<br>• *Legitimation*<br><br>*Collective action:*<br>• *Interactional workability*	• Trigger films demonstrate commonality and difference in experiences and facilitate former patients and carers identification as a group with shared beliefs on changing patients' activity experiences in their local stroke units.<br>• Trigger films are a powerful means of highlighting stroke survivors' and carers' experiences related to inactivity and activity in stroke units-these heighten staff understanding of stroke survivors' individual and collective experiences<br>• EBCD/AEBCD engages participants as equal partners in the improvement process.	• Recruitment and retention of a representative range of former patients & family members to participate in EBCD/AEBCD was challenging in some sites; numbers participating were higher at both London sites.
Small numbers of staff, volunteers, former patients, family members participate in co-design groups.	*Coherence:*<br>• *Communal specification*<br>• *Internalisation*<br><br>*Cognitive participation:*<br>• *Enrolment*<br>• *Legitimation*<br>• *Activation*<br><br>*Collective action:*<br>• *Interactional workability*<br>• *Relational integration*	• Co-design groups were effective with small numbers of regular participants<br>• Co-design groups tended to foster participants' continued commitment to individual and collective action and practice change;<br>• Co-design groups provided a mechanism to involve non stroke unit staff who could suggest and sanction improvements related to estates and community involvement;<br>• Co-design groups were the drivers of change and improvement	• Wider staff groups had less involvement in planning and actioning change planned by co-design groups;<br>• Staff numbers varied with many more nurses and HCSWs working in stroke teams than therapists, stroke SRAs and medical staff;<br>• Strategies for communicating planned change had varied success (newsletters, using routine staff information exchange events, open afternoons);<br>• Staff participants in co-design groups had increased workloads, typically with no additional time allocation.
Researcher facilitation of EBCD/AEBCD process	*Cognitive participation:*<br>• *Initiation*<br>• *Enrolment*<br>• *Legitimation*<br>• *Activation*<br><br>*Collective action:*	• Researcher facilitation of the structured EBCD/AEBCD enabled key staff to commit to participation in co-design groups and to developing and implementing improvements whilst maintaining their usual work roles and responsibilities;	• Researcher facilitation could mean some staff relied on researchers to lead and progress the EBCD/AEBCD process rather than assuming leadership roles;<br>• Time required to contact, engage with, involve and sustain participation of non stroke unit staff, volunteers and community groups was initially very high;

For peer review only - http://bmjopen.bmj.com/site/about/guidelines.xhtml

	• *Interactional workability*<br>• *Relational integration*<br>• *Skill set workability*	• Researcher facilitation was a key part of recruitment, retention and participation of former patients and carers;<br>• Researcher facilitation was important in enabling and sustaining involvement of wider hospital staff, volunteers and community involvement;<br>• Researcher facilitation supported key stroke unit staff in assuming responsibility for improvements after the CREATE study ended.	• Evidence in other EBCD projects of improvement activity stalling or ceasing when facilitator role ends.
The influence of managerial authority and support for stroke unit staff participation and improvement activity	*Cognitive participation:*<br>• *Initiation*<br>• *Enrolment*<br>• *Legitimation*<br><br>*Collective action:*<br>• *Interactional workability*<br>• *Relational integration*<br>• *Skill set workability*<br>• *Contextual integration*	• Managerial support for involvement of staff in the EBCD/AEBCD project was critical to the progression of the process in all sites-both in terms of joint meeting attendance for larger groups of staff and those staff routinely involved in co-design groups;<br>• Managerial support and clinical directorate and stroke unit level was required for co-design groups to navigate complex NHS Trust organisational structures and, in leveraging resources to enable co-design group ideas to progress;<br>• Involving managers with experience of service improvement elsewhere in the hospital in co-design groups ensured myths and misconceptions about what changes were possible in the organisation were quickly addressed and corrected.	• Securing direct involvement of non stroke unit managers in a project which did not directly address clinical outcomes or National Clinical Audit targets was challenging in all sites;<br>• Stroke unit staff were reluctant to take direct action involving estates to effect environmental support or use of volunteers on units in the absence of managerial support ay clinical directorate or stroke service level.
Challenges related to the duration of the change process and time taken for improvements to be introduced.	*Cognitive participation:*<br>• *Legitimation*<br>• *Activation*<br><br>*Collective action:*<br>• *Relational integration*<br>• *Skill set workability*<br>• *Contextual integration*<br><br>*Reflexive monitoring:*	• Full EBCD cycles took approximately 9 months to complete;<br>• AEBCD cycles took approximately 6 months to complete;	• Some seemingly simple improvements took many weeks to plan, develop and implement e.g. improved web access; making iPads accessible and secure; providing new and welcoming signage; facilitating on unit volunteer and community group activities;<br>• Improvements involving changes in space use, environment or volunteer involvement required negotiation with and sometimes additional funding from managers; this could take long periods of time or be blocked or ignored by managers.

For peer review only - http://bmjopen.bmj.com/site/about/guidelines.xhtml

	• *Systematisation*<br>• *Communal appraisal*<br>• *Individual appraisal*		
Implementing and embedding co-designed changes into stroke unit practice	*Collective action:*<br>• *Relational integration*<br>• *Skill set workability*<br>• *Contextual integration*<br><br>*Reflexive monitoring:*<br>• *Systematisation*<br>• *Communal appraisal*<br>• *Individual appraisal*	• 'Visible' improvements including new spaces for activity and environmental changes helped staff not involved in co-design groups understand the kinds of improvements occurring and led to inclusion of these spaces or environmental changes in the practice of therapists and nurses;<br>• Introduction and use of co-designed improvements such as the 'Something about me board' and personalising bed spaces with photos and personal objects were reported to prompt more personalised supervised and independent activity opportunities;<br>• Where new activity opportunities supported or could be added to existing rehabilitation practice these quickly became incorporated in the routines of therapists and nurses;<br>• Agreed, routine volunteer led activity was recognised as valuable in increasing social and cognitive activity opportunities for stroke survivors-this reduced demand on stroke unit staff and helped ensure volunteers had a defined role and contribution on the unit.	• Involving large and diverse staff groups as in stroke units in developing and implementing improvements proved challenging;<br>• Some staff felt they were not consulted about improvements which would require their on-going involvement e.g. completion of required information on the 'something about me boards'; preparing inpatients for and supporting them in breakfast club activity;<br>• Ensuring unit wide change , across disciplines, in encouraging and supporting activity opportunities was more difficult than securing uni-discipinary commitment to increasing activity opportunities;<br>• Routine volunteer activity on units was variably received-staff need to be confident volunteers have received appropriate stroke specific training for example if involved in communication support or mealtime support for stroke survivors.

For peer review only - http://bmjopen.bmj.com/site/about/guidelines.xhtml

Figure 1 Sites 1 and 2: EBCD

Stage 1: Setting up
Site set up, communications and awareness-raising with senior management and clinical teams
EBCD full day training for four staff from each site

Stage 2: Engaging staff and patients
Pre-implementation data collection + generation of trigger films:

Videoed semi-structured interviews with former patients and carers
Semi-structured interviews with staff
PROM / PREM
Non-participant observations
Behavioural mapping

Stage 3: Separate staff and patient/family events

Stage 4: Joint event
Patients, families, clinical staff view trigger films, share findings and set priorities for co-design

Stage 5: Small co-design teams
Space, *Activity* and *Communication* co-design groups meet (4-5 times per site)

Stage 6: Celebration event
Sharing improvements and plan for sustainability and further improvement

Post-implementation data collection:
Semi-structured interviews with former patients and carers
Semi-structured interviews with staff
PROM / PREM, Non-participant observations
Behavioural mapping

Sites 3 and 4: AEBCD

Stage 1: Setting up
Site set up, communications and awareness-raising with senior management and clinical teams
AEBCD full day training for four staff from each site

Stage 2: Engaging staff and patients
Pre-implementation data collection:
Semi-structured interviews with former patients and carers
Semi-structured interviews with staff
PROM / PREM
Non-participant observations
Behavioural mapping

Stage 3: Joint event
Patients, families, clinical staff to view trigger films generated by sites 1 and 2, share findings and set priorities for co-design

Stage 4: Small co-design teams
Space, *Activity* and *Communication* co-design groups meet (4-5 times per site)

Stage 5: Celebration event
Sharing improvements and plan for sustainability and further improvement

Post-implementation data collection:
Semi-structured interviews with former patients and carers
Semi-structured interviews with staff
PROM / PREM, Non-participant observations
Behavioural mapping

For peer review only

Supplementary File 1: Data collection linked to Normalisation Process Theory

**Focus for and use of Normalisation Process Theory in the process evaluation of the CREATE study. NB: the elements are not necessarily progressed through in a linear way; there is some degree of cyclical movement back and forth between elements.**			
**NPT element**	**Focus**	**Data collection (keep in NVivo or as word documents)**	**Notes**
**Coherence**	EBCD/AEBCD training for staff from sites	Researcher reflective notes on the training days<br><br>Point of Care Foundation training feedback sheets (anonymised) were available for the full day training and then feedback/post it notes from the half day training.	
	Informal staff comments and observations during: non-participant observations	Researchers' reflective notes on staff perceptions and understanding of the aims of the study (increase patient activity outside of therapy through co-designed interventions.	Include any evidence of access to the Kings Fund Toolkit, any evidence of dialogue between stroke unit teams (e.g. in handovers, at MDT or other staff meetings)
	Staff interviews pre interventions with:<br>a) EBCD/AEBCD champions at each site<br>b) Remaining staff interviewees	Interview transcripts (where these occurred)<br><br>Researchers' reflective notes on staff perceptions and understanding of the aims of the study and any evidence of actions to involve the wider stroke unit team<br>Researchers' reflective notes on staff perceptions and understanding of the aims of the study<br>Review documentary evidence (notes of meetings/action plans/reviews of action plans)	(Overlaps with cognitive participation, collective action and reflexive monitoring)
	Study Oversight Groups (SoG) meetings	SoG members at sites include: Deputy Directorate Manager, Therapy Lead (Manager), Service Improvement Lead, Volunteer Manager, Stroke Specialist Nurse.	These were largely virtual meetings - review notes and email trails/responses from any SoG members

Supplementary File 1: Data collection linked to Normalisation Process Theory

**Cognitive participation**	Staff interviews post interventions with:<br>a) EBCD/AEBCD champions at each site<br>b) Remaining staff interviewees	Interview transcripts<br><br>Researchers' reflective notes on staff perceptions and understanding of the aims of the study and any evidence of actions to involve the wider stroke unit team	***Overlaps with coherence***
	Preparation for and conduct of staff event and patient event and joint event	Patient and staff questionnaire* feedback<br>EBCD champions individual reflective summary (Guide given)<br>Researchers' reflective notes on staff perceptions and understanding of the aims of the study and any evidence of actions to involve the wider stroke unit team	Questionnaires and reflective summary sheets adapted from those used in the Locock et al (2014) study
	Co-design group meetings	Patient and staff questionnaire feedback<br>EBCD champions individual reflective summary<br>Review documentary evidence (notes of meetings/action plans/reviews of action plans)<br>Researchers' reflective notes on staff perceptions and understanding of the co-design work and any evidence of actions to involve the wider stroke unit team.	As above- seek questionnaires and reflective summaries after each event or meeting.
	Study Oversight Groups (SoG) any ongoing meetings	Researchers' reflective notes on staff perceptions and understanding of the progress of the study and of staff and patient involvement in this. Review documentary evidence (notes of meetings/action plans/reviews of action plans)	

For peer review only

Supplementary File 1: Data collection linked to Normalisation Process Theory

**Collective action**	Co-design groups<br>And implementation activity<br><br>And feedback from the celebratory events at both sites	Any patient and staff questionnaire* feedback<br>EBCD champions individual reflective summary<br>Review documentary evidence (notes of meetings/action plans/reviews of action plans)<br><br>Researchers' reflective notes on staff's and patients' and carers' engagement with the co-design work and evidence of actions to involve the wider stroke unit team. (E.g. be alert to discussion at MDT or other meetings), evidence of impacts on existing work of staff and how this is managed/discussed.<br><br>Researchers' reflective notes on range of interventions/activities introduced and how these are managed by individuals and groups (e.g. therapists/nurses/volunteers) and whether and how these are publicised by the EBCD champions or others	
**Reflexive monitoring**	Co-design groups<br>And implementation activity<br><br>Staff interviews with:<br>a) EBCD/AEBCD champions at each site<br>b) Remaining staff<br><br>Patient and carer interviews<br><br>Informal staff comments and observations during: non-participant observations	Data collection as above<br><br>Interview transcripts<br><br>Researchers' reflective notes on staff perceptions of the study and how the co-design work and interventions have affected patients' activity levels and overall inpatient experience.<br>Review the involvement of the wider stroke unit team in interventions to increase activity levels<br><br>Review documentary evidence (notes of meetings/action plans/reviews of action plans)<br><br>Interview transcripts<br><br>Researchers' reflective notes on staff perceptions and understanding of the outcomes of the study (increase patient activity outside of therapy through co-designed interventions.	**Links back to Cognitive participation**- review reflexive monitoring activity at the when co-design groups are established and as interventions/activities are piloted

For peer review only - http://bmjopen.bmj.com/site/about/guidelines.xhtml

Supplementary File 1: Data collection linked to Normalisation Process Theory

**Additional focus on:**	Absence of separate staff and patient meetings in AEBCD sites<br><br>Shortened timescale for co-design at AEBCD sites<br><br>Differences in level of patient and carer recruitment for EBCD/AEBCD groups between sites.	Consider the impact on the joint meeting and progression to co-design work<br><br>Examples are numbers of people involved different sites.	
**Organisational factors impacting on set up of CREATE at sites:**	Consider: managerial involvement or its absence, project disruption at sites interest and support at the wider organisational level or beyond	Consider other organisational factors relevant to the conduct of the study at each site including:<br><br>Key staff absence or key staff leaving the organisation<br><br>Ongoing or specific staffing problems<br><br>Positive/innovative factors such as the fundraising and design competition at site 3.<br><br>And the pre-existing funding for day room refurbishment at Site 2, and the ongoing Rooting and Fruiting gardening project at Site 4	

For peer review only - http://bmjopen.bmj.com/site/about/guidelines.xhtml

Supplementary file 2: Demographic data all participants

Patient demographics

**Site 1 Patient interviews (n= 9) pre-implementation of EBCD**	
**Age**	**Gender**
65 +/-12 years	M = 5<br>F = 4
**Site 1 Patient interviews (n= 5) post-implementation of EBCD**	
65 +/-13 years	M = 3<br>F = 2
**Site 2 Patient interviews (n= 9) pre-implementation of EBCD**	
68 =/- 18 years	M= 5<br>F= 4
**Site 2 Patient interviews (n=6) post-implementation of EBCD**	
73 +/- 12 years	M =6
**Site 3 Patient interviews (n= 9) pre-implementation of EBCD**	
61 +/- 16 years	M = 5<br>F = 4
**Site 3 Patient interviews (n= 6) post-implementation of EBCD**	
67 +/- 18 years	M = 4<br>F = 2
**Site 4 Patient interviews (n= 4) pre-implementation of EBCD**	
72 +/- 7 years	M = 3<br>F = 1
**Site 4 Patient interviews (n= 6) post-implementation of EBCD**	
68 +/- 10 years	M = 2<br>F = 4

F female, M male

Family carer demographics

**Site 1 Carer interviews (n= 4) pre-implementation of EBCD**	
Age	Gender
45-54	F
18-24	M
55-64	M
55-64	F
**Site 1 Carer interviews (n= 5) post-implementation of EBCD**	
18-24	M
55-64	M
45-54	F
65-74	M
75-84	F

For peer review only

**Site 2 Carer interviews (n=4) pre-implementation of EBCD**	
55-64	F
65-74	F
65-74	F
75-84	M
**Site 2 Carer interviews (n= 2) post-implementation of EBCD**	
75-84	F
75-84	F
**Site 3 Carer interviews (n=3) pre-implementation of AEBCD**	
65-74	F
55-64	F
75-84	F
**Site 3 Carer interviews (n=3) post-implementation of AEBCD**	
65-74	F
65-74	M
18-24	F
**Site 4 Carer interviews (n=2) pre-implementation of AEBCD**	
55-64	F
65-74	M
**Site 4 Carer interviews (n=5) post-implementation of AEBCD**	
85-94	M
65-74	M
55-64	M
65-74	M
55-64	F

Demographic data staff participants

**Site 1 Staff interviews (n=13) pre-implementation of EBCD**	
**Age**	**Profession**
45-54	Doctor
25-34	RN
35-44	RN
35-44	HCSW
45-54	HCSW
45-54	Domestic
35-44	OT
25-34	OT
45-54	PT
25-34	PT
35-44	Clinical Psychologist
25-34	SALT
35-44	Dietician
**Site 1 Staff interviews (n=8) post-implementation of EBCD**	
25-34	SALT
25-34	PT
35-44	OT
35-44	Dietician
35-44	SRA
35-44	Operations manager
25-34	RN
45-54	Consultant
**Site 2 Staff interviews (n=15) pre-implementation of EBCD**	
35-44	PT
25-34	PT
18-24	SRA
25-34	HCSW

For peer review only

35-44	SALT
35-44	OT
25-34	Dietician
25-34	OT
25-34	RN
25-34	RN
25-34	RN
55-64	Volunteer
25-34	Dietician
35-44	HCSW
35-44	Doctor
**Site 2 staff interviews (n= 7) post-implementation of EBCD**	
25-34	RN
25-34	PT
35-44	HCA
25-34	SRA
25-34	RN
25-34	OT
45-54	HCSW
**Site 3 Staff interviews (n= 6) pre-implementation of AEBCD**	
35-44	Clinical Psychologist
35-44	OT
25-34	PT
35-44	RN
45-54	PT
45-54	Doctor
**Site 3 Staff interviews (n= 8) post-implementation of AEBCD**	
35-44	Clinical Psychologist
25-34	OT
25-34	PT
45-54	RN
45-54	PT
45-54	Doctor
35-44	HCSW
35-44	SRA
**Site 4 Staff interviews (n= 7) pre-implementation of AEBCD**	
35-44	TA
35-44	Doctor
45-54	RN
25-34	PT
25-34	OT
55-64	RN
45-54	SALT
**Site 4 Staff interviews (n= 9) post-implementation of AEBCD**	
25-34	SALT
35-44	RN
25-34	HCSW
25-34	PT
55-64	RN
45-54	RN
25-34	OT
45-54	TA
35-44	RN

PT physiotherapist, OT, occupational therapist, SALT speech and language therapist, RN, registered nurse, SRA stroke rehabilitation assistant, TA therapy assistant, HCSW healthcare support worker.

For peer review only

Supplementary file 3: Number of Co-design groups held

	Co-design participants			Number of co-design group meetings
	Stroke survivors	Carers	Staff (includes volunteers)	
Site 1 (EBCD)	5	6	13	10
Site 2 (EBCD)	4	4	12	9
Site 3 (AEBCD)	11	7	14	15
Site 4 (AEBCD)	3	4	15	9
**TOTAL**	**23**	**21**	**54**	**43**

For peer review only

COREQ (COnsolidated criteria for REporting Qualitative research) Checklist

A checklist of items that should be included in reports of qualitative research. You must report the page number in your manuscript where you consider each of the items listed in this checklist. If you have not included this information, either revise your manuscript accordingly before submitting or note N/A.

Topic	Item No.	Guide Questions/Description	Reported on Page No.
**Domain 1: Research team and reflexivity**			
Personal characteristics			
Interviewer/facilitator	1	Which author/s conducted the interview or focus group?	
Credentials	2	What were the researcher's credentials? E.g. PhD, MD	
Occupation	3	What was their occupation at the time of the study?	
Gender	4	Was the researcher male or female?	
Experience and training	5	What experience or training did the researcher have?	
Relationship with participants			
Relationship established	6	Was a relationship established prior to study commencement?	
Participant knowledge of the interviewer	7	What did the participants know about the researcher? e.g. personal goals, reasons for doing the research	
Interviewer characteristics	8	What characteristics were reported about the inter viewer/facilitator? e.g. Bias, assumptions, reasons and interests in the research topic	
**Domain 2: Study design**			
Theoretical framework			
Methodological orientation and Theory	9	What methodological orientation was stated to underpin the study? e.g. grounded theory, discourse analysis, ethnography, phenomenology, content analysis	
Participant selection			
Sampling	10	How were participants selected? e.g. purposive, convenience, consecutive, snowball	
Method of approach	11	How were participants approached? e.g. face-to-face, telephone, mail, email	
Sample size	12	How many participants were in the study?	
Non-participation	13	How many people refused to participate or dropped out? Reasons?	
Setting			
Setting of data collection	14	Where was the data collected? e.g. home, clinic, workplace	
Presence of non-participants	15	Was anyone else present besides the participants and researchers?	
Description of sample	16	What are the important characteristics of the sample? e.g. demographic data, date	
Data collection			
Interview guide	17	Were questions, prompts, guides provided by the authors? Was it pilot tested?	
Repeat interviews	18	Were repeat inter views carried out? If yes, how many?	
Audio/visual recording	19	Did the research use audio or visual recording to collect the data?	
Field notes	20	Were field notes made during and/or after the inter view or focus group?	
Duration	21	What was the duration of the inter views or focus group?	
Data saturation	22	Was data saturation discussed?	
Transcripts returned	23	Were transcripts returned to participants for comment and/or	

For peer review only

Topic	Item No.	Guide Questions/Description	Reported on Page No.
		correction?	
**Domain 3: analysis and findings**			
Data analysis			
Number of data coders	24	How many data coders coded the data?	
Description of the coding tree	25	Did authors provide a description of the coding tree?	
Derivation of themes	26	Were themes identified in advance or derived from the data?	
Software	27	What software, if applicable, was used to manage the data?	
Participant checking	28	Did participants provide feedback on the findings?	
Reporting			
Quotations presented	29	Were participant quotations presented to illustrate the themes/findings? Was each quotation identified? e.g. participant number	
Data and findings consistent	30	Was there consistency between the data presented and the findings?	
Clarity of major themes	31	Were major themes clearly presented in the findings?	
Clarity of minor themes	32	Is there a description of diverse cases or discussion of minor themes?	

Developed from: Tong A, Sainsbury P, Craig J. Consolidated criteria for reporting qualitative research (COREQ): a 32-item checklist for interviews and focus groups. *International Journal for Quality in Health Care*. 2007. Volume 19, Number 6: pp. 349 – 357

Once you have completed this checklist, please save a copy and upload it as part of your submission. DO NOT include this checklist as part of the main manuscript document. It must be uploaded as a separate file.

For peer review only

GRIPP2 short form

Section and topic	Item	Reported on page No
1: Aim	Report the aim of PPI in the study	4-5
2: Methods	Provide a clear description of the methods used for PPI in the study	4-5,6,8-15
3: Study results	Outcomes—Report the results of PPI in the study, including both positive and negative outcomes	15-17
4: Discussion and conclusions	Outcomes—Comment on the extent to which PPI influenced the study overall. Describe positive and negative effects	15-17
5: Reflections/critical perspective	Comment critically on the study, reflecting on the things that went well and those that did not, so others can learn from this experience	15-17

PPI: patient and public involvement.

From: Staniszewska, S., Brett, J., Simera, I. *et al.* GRIPP2 reporting checklists: tools to improve reporting of patient and public involvement in research. *Res Involv Engagem* **3,** 13 (2017). https://doi.org/10.1186/s40900-017-0062-2

For peer review only

BMJ Open

Co-designing organisational improvements and interventions to increase inpatient activity in four stroke units in England; a mixed-methods process evaluation using Normalisation Process Theory.

Journal:	*BMJ Open*
Manuscript ID	bmjopen-2020-042723.R1
Article Type:	Original research
Date Submitted by the Author:	25-Nov-2020
Complete List of Authors:	Clarke, David; University of Leeds Faculty of Medicine and Health, Academic Unit of Ageing and Stroke Research, Leeds Institute of Health Sciences<br>Gombert-Waldron, Karolina; Kingston University & St George’s, University of London, Faculty of Health and Social Care Sciences<br>Honey, Stephanie; University of Leeds, Leeds Institute of Health Sciences<br>Cloud, Geoffrey; Monash University Melbourne and Alfred Health, Department of Clinical Neuroscience, CCS.<br>Harris, Ruth; King\'s College London<br>Macdonald, Alastair; The Glasgow School of Art<br>McKevitt, Christopher; King's College London, Department of Public Health Sciences<br>Robert, Glenn; King's College London<br>Jones, Fiona; St Georges University of London
**Primary Subject Heading**:	Rehabilitation medicine
Secondary Subject Heading:	Health services research
Keywords:	Stroke < NEUROLOGY, REHABILITATION MEDICINE, Quality in health care < HEALTH SERVICES ADMINISTRATION & MANAGEMENT, QUALITATIVE RESEARCH

SCHOLARONE™
Manuscripts

I, the Submitting Author has the right to grant and does grant on behalf of all authors of the Work (as defined in the below author licence), an exclusive licence and/or a non-exclusive licence for contributions from authors who are: i) UK Crown employees; ii) where BMJ has agreed a CC-BY licence shall apply, and/or iii) in accordance with the terms applicable for US Federal Government officers or employees acting as part of their official duties; on a worldwide, perpetual, irrevocable, royalty-free basis to BMJ Publishing Group Ltd ("BMJ") its licensees and where the relevant Journal is co-owned by BMJ to the co-owners of the Journal, to publish the Work in this journal and any other BMJ products and to exploit all rights, as set out in our licence.

The Submitting Author accepts and understands that any supply made under these terms is made by BMJ to the Submitting Author unless you are acting as an employee on behalf of your employer or a postgraduate student of an affiliated institution which is paying any applicable article publishing charge ("APC") for Open Access articles. Where the Submitting Author wishes to make the Work available on an Open Access basis (and intends to pay the relevant APC), the terms of reuse of such Open Access shall be governed by a Creative Commons licence – details of these licences and which Creative Commons licence will apply to this Work are set out in our licence referred to above.

Other than as permitted in any relevant BMJ Author's Self Archiving Policies, I confirm this Work has not been accepted for publication elsewhere, is not being considered for publication elsewhere and does not duplicate material already published. I confirm all authors consent to publication of this Work and authorise the granting of this licence.

For peer review only

Title:

Co-designing organisational improvements and interventions to increase inpatient activity in four stroke units in England; a mixed-methods process evaluation using Normalisation Process Theory

Authors:

David J Clarke[1], Karolina Gombert-Waldron[2], Stephanie Honey[1], Geoffrey C Cloud[3], Ruth Harris[4], Alastair Macdonald[5], Christopher McKevitt[6], Glenn Robert[4], Fiona Jones[2].

[1]Leeds Institute of Health Sciences. University of Leeds, UK
[2]Faculty of Health and Social Care Sciences. Kingston University & St George's, University of London. UK.
[3] Department of Clinical Neuroscience, CCS, Monash University Melbourne and Alfred Health, Melbourne, Australia.
[4]Florence Nightingale Faculty of Nursing, Midwifery & Palliative Care, King's College London. UK.
[5]School of Design, Glasgow School of Art. UK.
[6]School of Population Health & Environmental Sciences, Faculty of Life Sciences and Medicine, King's College London. UK.

Corresponding author: **David J Clarke**
Corresponding author contact details:
d.j.clarke@leeds.ac.uk ORCID ID: 0000-0001-6279-1192

Word count: 6253

For peer review only - http://bmjopen.bmj.com/site/about/guidelines.xhtml

Abstract

Objective: To explore facilitators and barriers to using Experience-based Co-design (EBCD) and accelerated EBCD (AEBCD) in the development and implementation of interventions to increase activity opportunities for inpatient stroke survivors.

Design: Mixed-methods process evaluation underpinned by normalisation process theory (NPT).

Setting: Four post-acute rehabilitation stroke units in England.

Participants: Stroke survivors, family members, stroke unit staff, hospital managers, support staff and volunteers. Data informing our NPT analysis comprised: ethnographic observations, n= 366 hours; semi-structured interviews with 76 staff, 53 stroke survivors and 27 family members pre or post EBCD/AEBCD implementation; and observation of 43 co-design meetings involving 23 stroke survivors, 21 family carers and 54 staff

Results: Former patients and families valued participation in EBCD/AEBCD perceiving they were equal partners in co-design. Staff engaged with EBCD/AEBCD, reporting it as a valuable improvement approach leading to increased activity opportunities. The structured EBCD/AEBCD approach was influential in enabling coherence and cognitive participation and legitimated staff involvement in the change process. Researcher facilitation of EBCD/AEBCD supported cognitive participation, collective action and reflexive monitoring; these were important in implementing and sustaining co-design activities. Observations and interviews post EBCD/AEBCD cycles confirmed creation and use of new social spaces and increased activity opportunities in all units. EBCD/AEBCD facilitated engagement with wider hospital resources and local communities, further enhancing activity opportunities. However, outside of structured group activity many individual staff-patient interactions remained task-focused.

Conclusions: EBCD/AEBCD facilitated the development and implementation of environmental changes and revisions to work routines which supported increased activity opportunities in stroke units providing post-acute and rehabilitation care. Former stroke patients and carers contributed to improvements. NPT's generative mechanisms were instrumental in analysis and interpretation of facilitators and barriers at the individual, group and organisational levels and can help inform future implementations of similar approaches.

Strengths and limitations of this study:

- This process evaluation reports the first use of Experience-based Co-design (EBCD) and accelerated EBCD (AEBCD) in stroke services.
- Analysis informed by normalisation process theory before, during and after use of EBCD/AEBCD provided for an in-depth understanding of staff engagement, local organisational contexts, the impact of co-designed changes on day-to-day working practices of stroke unit staff and the experiences of stroke survivors both as inpatients and as participants in EBCD/AEBCD.
- Recruitment of stroke survivors and family members to participation in EBCD/AEBCD activities was good across all sites but it proved more difficult to recruit former inpatient stroke survivors to participate in post EBCD/AEBCD evaluation interviews.
- The process evaluation was not designed to generate data to evaluate the longer-term sustainability of interventions developed to increase activity opportunities for inpatient stroke survivors.
- Researchers undertaking the process evaluation were part of the core research team for the CREATE study and not a separately employed process evaluation team.

For peer review only - http://bmjopen.bmj.com/site/about/guidelines.xhtml

Introduction

Stroke is the second most common cause of death worldwide[1] and is associated with significant long-term disability[2]. Specialist rehabilitation in stroke units contributes substantially to regaining independence[3]. However, observational studies in stroke units identify high levels of inactivity. In studies spanning forty years, patients have been reported to be physically active between 13% and 23% of the waking day, and engaged in cognitive or social activity between 4% and 32% of the waking day[4-10]. In these studies, activity related largely to participation in planned therapy, typically physiotherapy (PT), occupational therapy (OT), speech and language therapy (SLT)[11-14]. Outside of planned therapy patients report being bored and can be alone and inactive for 60% of the day[7,15-16].

This seemingly intractable problem was addressed in two early studies in England. Following observation of activity levels across four rehabilitation wards for the elderly Ellul et al (1993) introduced individualised (physical) activity programmes on two wards facilitated by nurses and scheduled social group activities with staff acting as activity leads; in total 51 patients were observed pre and post-intervention[17].Similarly, in a single rehabilitation unit across a period of two years Newell et al (1997) introduced regular leisure activity service visits; access to computers, weekly discussion groups, communal lunches and group meetings with other stroke survivors, and encouraged family involvement in therapy practice; activity levels of 67 patients were observed during this time period.[18]. Both studies reported increases in useful activity and decreased time spent passively at the bedside. However, neither study led to wider adoption of the approaches reported. Whilst post-stroke inactivity can contribute to poorer outcomes, increased participation in structured physical activity is associated with improved physical function[19] and greater independence[20]. Evidence suggests increasing social and cognitive activity may reduce burden associated with post-stroke mood disorders and cognitive impairment[21]. Interest is increasing in the potential of environmental enrichment (EE)[8,10,22-25] to increase physical, social and cognitive activity in stroke units. EE is defined as *'interventions designed to facilitate physical (motor and sensory), cognitive and social activity by provision of equipment and organisation of a stimulating environment'* [23]p48. There is also interest in the impact of the built environment on activity and social interaction[26-27] . However, across these studies, there remains limited evidence of sustained change in inpatient activity [22-25].

To date, approaches to address inactivity post-stroke have been largely externally designed and researcher led with limited or no involvement of stroke survivors, caregivers or staff in study design or delivery. As an alternative, participatory improvement approaches involve directly engaging service

For peer review only - http://bmjopen.bmj.com/site/about/guidelines.xhtml

users and providers in a collaborative process to 'co-produce' a service that addresses the needs and wants of stakeholders whilst ensuring the improved service can be delivered utilising existing resources[28-32]. Co-production approaches provide a means for stakeholder voices not only to be heard in terms of improving services but also provide a framework for stakeholder participation throughout the improvement process[29,32-34]. Specific forms of co-production such as Experience-based Co-design (EBCD)[29,35-37] have been developed and applied in healthcare. The Collaborative Rehabilitation in Acute Stroke (CREATE) study evaluated the impact of using the six-stage EBCD approach in two stroke units and an accelerated version (AEBCD)[38-39] in two further units (Figure 1) to increase patients' social, cognitive and physical activity. The CREATE study findings are reported elsewhere[38-39]; in summary, qualitative findings indicated it was feasible to co-produce changes in all four stroke units to increase opportunities for social, cognitive and physical activity through joint work in three priority areas: 'Space' (environment), 'Activity' and 'Communication'. Patients, families and staff perceived positive benefits from participating in EBCD/AEBCD. However, quantitative data did not demonstrate consistent increases in physical, social or cognitive activity.

Despite extensive use of EBCD internationally in the last ten years, there has been limited evaluation of the process and outcomes of the approach, and none in stroke[36,40]. The use of EBCD/AEBCD in the CREATE study to develop and implement quality improvements is typical of a complex intervention involving '*multiple components which interact to produce change*'[41]. Medical Research Council[41-42] guidance for evaluation of complex interventions recommends use of process evaluations to explore not only '*whether interventions 'worked' but [also] how they were implemented, their causal mechanisms and how effects differed from one context to another*'[42]. This paper reports on the embedded process evaluation in the CREATE study. The aim of the process evaluation was to explore facilitators and barriers to using EBCD and AEBCD in the development and implementation of improvements to increase activity opportunities for inpatient stroke survivors.

Insert Figure 1: *CREATE study design and methods with embedded process evaluation about here*

Methods:

Patient and Public Involvement:

Listening to and utilising patients' and carers' voices, experiences and ideas is central to EBCD[29] but patients and the public were also involved from study inception, participating in development of the

For peer review only - http://bmjopen.bmj.com/site/about/guidelines.xhtml

research proposal which was discussed with stroke survivors and carers at two Stroke Research group meetings. There was strong support for the research, particularly the participatory approach planned; these groups provided important insights about how to facilitate participants' involvement including being aware of post-stroke fatigue, challenges of access to hospitals sites, running EBCD/AEBCD events on or near to stroke units and ensuring transport, parking, access and expenses for involvement were considered in advance of onsite participation. A younger stroke survivor and carer became members of the Study Steering Committee (SSC), participating in review of participant information, discussions about conducting observations, interviews and co-design meetings with patients and staff. They helped shape the messaging in EBCD/AEBCD feedback events, reviewed emerging findings and commented on researchers' summary explanations of findings.

CREATE study design and methods

CREATE used a mixed-methods case-comparison design with embedded process evaluation[38-39] (Figure 1). EBCD[29,31,35] or AEBCD[37] was introduced into four stroke units (sites 1-4), two in London and two in the North of England (Yorkshire).

The Health Research Authority and South East Coast Brighton & Sussex Research Ethics Committee (16/LO/0212) provided ethical approval. Written site consent for data collection was provided by the Principal Investigator at each site. For non-participant observations we used a verbal process consent approach, checking that staff, family carers and stroke survivors agreed to observations; no individual data were recorded. We gained written informed consent from all stroke survivors, family carers and staff participating in interviews and EBCD/AEBCD activities.

Contexts

Stroke units were purposively selected, focusing on units not already taking part in large clinical trials, that could commit to participation in a multi-stage study over 6-9 months. Sites were stroke units receiving patients after care in hyper-acute units in the same hospital (sites 2 and 4) or major stroke centres (sites 1 and 3) Table 1. Biannual national Acute Stroke Organisational audit data[43] indicated the units performed within the mid-range across key quality indicators and were subject to the same staffing pressures and increasing caseload complexity reported nationally. Clinical leads at each site acted as Principal Investigators or supported the study.

Insert Table 1: Unit characteristics about here

For peer review only - http://bmjopen.bmj.com/site/about/guidelines.xhtml

For peer review only

Participants

As part of the embedded process evaluation semi-structured interviews were conducted with 76 staff, 53 stroke survivors (previously inpatients) and 27 family carers pre or post EBCD/AEBCD implementation. 43 co-design meetings were held across sites involving 23 stroke survivors, 21 family carers and 54 staff including PTs , OTs , SLTs , dieticians, nurses, rehabilitation and healthcare support workers (HCSW), hospital managers, support staff and volunteers. 366 hours of ethnographic observations were completed (Table 2).

Insert Table 2: Participants about here

Process Evaluation

Normalisation Process Theory (NPT)[44-45] underpinned the process evaluation which encompassed sites preparation for and use of EBCD/AEBCD. NPT is concerned with understanding how complex interventions are implemented and integrated into existing healthcare systems and is conceptualised through four generative mechanisms each with four components (Table 3). These mechanisms represent what participants 'do' to get the work of implementation done. They can be understood as a process (not necessarily linear) in which participants make sense of a new or different way of working, commit to it, make the effort required to work in that way and undertake continuous evaluation. NPT was used in two ways, firstly to guide data generation at each site and secondly as a sensitising lens in ongoing data analysis. NPT's constructs were used to identify and reflect upon processes that may act as facilitators or barriers to using EBCD/AEBCD to develop and implement improvements to increase activity opportunities for inpatient stroke survivors.

***Insert Table 3** Normalisation Process Theory about here*

Process evaluation data sources

A data generation plan linked to NPT's constructs identified data to be generated pre and post EBCD/AEBCD processes in sites, (Supplementary file 1). In each site data were generated by researchers pre and post-implementation of EBCD/AEBCD cycles through ethnographic non-participant observations of patient and staff activity at different times of the day and at weekends. Observations were conducted over a period of ten days, in sites 1-3 by FJ, KGW and AC, in sites 2 and 4 by DJC and SH. Observation periods typically lasted 4-5 hours; each researcher completed 3-4

For peer review only - http://bmjopen.bmj.com/site/about/guidelines.xhtml

observation periods in each unit pre and post-implementation. Semi-structured audio or video interviews were conducted by FJ,TK, AC and KGW insites 1 and 3 and by SH and DJC in sites 2 and 4 with a purposive sample of stroke unit staff, stroke survivors who had been inpatients in the units in the six months prior to EBCD/AEBCD cycles and family carers. Interviews were repeated post EBCD/AEBCD implementation. Researchers' observations of staff training in EBCD/AEBCD, reflections on facilitating EBCD/AEBCD cycles, and on informal and formal engagement with participants in sites as part of recruitment activity and through observations and interviews were included. See Supplementary file 2 for demographic data.

Process evaluation data analysis

Data analysis

Qualitative data were managed in NVivo 10[46] Using a process of on-going integrative analysis [47], themes were identified and reviewed at each site and then discussed FJ, DJC,KWG,SH in 3 monthly face-to-face meetings, followed by review of the full data set. Analysis was underpinned by use of NPT's constructs and subcomponents. Once EBCD/AEBCD activities ceased in all sites, summary memos with researcher reflections were used to construct a single integrated account. Confirmability occurred through independent, then joint and team half-day analysis cycles, followed by discussing emerging findings with SSC members. Credibility and transferability are evident in the use of data extracts to support explanations of observational and interview data in terms of participants' engagement with EBCD/AEBCD, as well as the facilitators and barriers to developing and implementing increased activity opportunities in sites.

Process evaluation results

Data generated through non-participant observations, semi-structured interviews and evaluations from EBCD/AEBCD events indicated positive experiences of participation in EBCD/AEBCD. Most participants perceived EBCD/AEBCD processes led to changes which were increasing opportunities for independent and supervised activity in all sites. These changes affected not only inpatient stroke survivors' experiences but also those of their family carers and stroke unit staff. NPT mechanisms rarely operate in a linear or standalone way, in table 4 and below we highlight the combinations of mechanisms we identified occurring within and across sites during the EBCD/AEBCD process and which in turn were identified as directly and indirectly helping to increase activity opportunities.

Insert table 4 about here:

Coherence and cognitive participation: making sense of and engaging with EBCD/AEBCD over time

For peer review only - http://bmjopen.bmj.com/site/about/guidelines.xhtml

Prior to research activity, members of each stroke team attended EBCD/AEBCD training led by the Point of Care Foundation[28]. These staff, supported by a researcher, were to lead EBCD/AEBCD implementation at their site. Full day training for sites 1 and 2 introduced the CREATE study and the theory and practice of EBCD/AEBCD; sites 3 and 4 received half-day training focused on implementation. Participants in AEBCD sites heard first-hand of how sites 1 and 2 were working to increase activity and discussed changes made by these teams.

> *"it was helpful meeting other people involved in the study on their wards, what they did and how it involved patients" (AEBCD training participant feedback site 3).*

Training afforded these staff the opportunity to start making sense of EBCD/AEBCD and to think individually and collectively about sharing their knowledge and understanding with colleagues, about what activity opportuntities were possible and what these would entail practically for the day-to-day work of their colleagues and themselves. Training enabled reflection on work with patients and carers using EBCD/AEBCD and how it differed from written feedback about patients' and carers' experiences. However, ensuring other stroke unit staff understood and engaged with the EBCD/AEBCD process was more challenging. There was no requirement for cascade training or other forms of knowledge sharing. In addition, changes to United Kingdom Health Research Authority (HRA) and Research Ethics Committee (REC) approvals processes led to a delay of four months between training site 1 and 2 staff and commencing EBCD activity, early interest quickly dissipated; understanding of what the EBCD approach would mean for wider staff groups' day-to-day work was limited.

However, researcher presence during pre-EBCD/AEBCD observation and interview periods enabled wider groups of staff to engage with and make sense of the project. Staff were curious about data generation methods used including behavioural mapping and observations, researchers used these opportunities to explain EBCD/AEBCD. Patients and carers also became aware of the study through the conduct of observations and behavioural mapping; they sought study information and expressed views on activity outside of therapy.

Coherence, cognitive participation and collective action: the faciliated EBCD/AEBCD process

In sites 1 and 2 EBCDs structured and facilitated approach was a major factor contributing to staff progressing to a more engaged position, readier to commit to considering how change could happen in their site and to thinking through who needed to be involved. Separate and then joint- meetings enabled large numbers of staff to participate in EBCD events (Figure 1). They viewed trigger films, heard first-hand accounts of patients' and carers' experiences, worked in small groups with colleagues, former patients and carers to identify 'touch points' and agreed priorities for action. These were high-energy

For peer review only - http://bmjopen.bmj.com/site/about/guidelines.xhtml

meetings with shared enthusiasm for change, and proved a powerful catalyst for larger groups of staff to share the view that change to stroke unit environments, access to resources, and routine-working practices to increase patient activity was possible. These events led to understanding of the role of co-design groups as being to work on ideas for activity opportunities generated by participants in joint-meetings. In sites 3 and 4, starting the AEBCD process at the later joint-meeting stage meant knowledge and understanding was more limited initially. However, joint-meetings still proved important in engaging staff, particularly as information on how sites 1 and 2 had addressed issues identified in trigger films were shared at these meetings.

"It felt quite exciting - it will be interesting to see how it develops, keen to be involved and contribute" (Feedback after Joint event, staff, site 3)

Other features of EBCD/AEBCD that facilitated cognitive participation and collective action, mainly in co-design group members, were the defined and time limited nature of the EBCD/AEBCD process. Clinical leads and service managers understood EBCD/AEBCD was a tried and tested service improvement model, this legitimised staff time and resource allocation committed to EBCD/AEBCD and associated improvements. Staff in all sites noted EBCD/AEBCD contrasted with previous attempts to introduce change, which were often 'poorly defined' in terms of timescales, roles and responsibilities and operated without additional resources. EBCD/AEBCD's participatory approach appeared to add a sense of responsibility for staff to deliver on agreed actions, and not to 'let down' patients and carers they worked with in co-design groups. As commitment to increasing activity opportunities grew, small groups of staff, not all of whom were stroke unit based, worked together to progress actions agreed by co-design groups.

Cognitive participation and collective action: patient and family member participation in EBCD/AEBCD

EBCD/AEBCD ensures patients and carers express their priorities for change and engages them as equal partners in designing and implementing solutions. In sites 1 and 2 separate patient and carer meetings provided opportunities to explore experiences of stroke and activity/inactivity after stroke. Trigger films demonstrated commonality and difference in experiences and began the process of identification by these former patients and carers as a group, with a shared belief in changing patients' activity experiences in their local stroke units. Although former patients and carers in sites 3 and 4 did not have local trigger film participation in common they recognised long periods of inactivity described in the site 1 and 2 trigger films and lack of activity opportunities as similar to their own; trigger films validated important issues raised. EBCD/AEBCD provided former patients and carers with the means and confidence to give voice to their experiences and appeared to help these participants understand how they could work with staff as partners in bringing about improvements.

For peer review only - http://bmjopen.bmj.com/site/about/guidelines.xhtml

"We felt able to say what we wanted to say and what we wanted to say has turned out to have a valuable effect, so, yeah, very happy to say that we didn't feel intimidated in any way" (Carer-Site 4, post).

Participants in EBCD/AEBCD shared a common experience with their peers and, with the staff with whom they exchanged ideas and problem solved in co-design groups. This also appeared true for managers, professional leads and volunteers who were 'external' to the day-to-day work of stroke units but involved in EBCD/AEBCD events. Patients' voices and the authentic examples depicted in trigger films were critical in all staff reorientating their focus to patients' experiences. These events contributed to participants' progression from shared understanding of the importance of change to working together to define ways to implement ideas into existing working practices. This included thinking through how changes to increase activity would be actioned, who would take responsibility and, who would routinely deliver these actions. These mechanisms represent progression through cognitive participation and collective action over time. Clinical staff facilitated all co-design groups, patients or carers did not seek to lead; but their contributions were actively pursued, and observational and interview data findings confirm these were influential in improvements that occurred. Examples included designing and painting murals (sites 1 and 3) and redesign of patient and carer facing documentation (sites 2 and 4), actions designed to facilitate increased activity through providing stimulating spaces and information about the purpose of new spaces and activity opportunities.

Cognitive participation and collective action: wider staff groups have less involvement in planning and actioning change

Co-design groups brought together those who believed in and were committed to making change happen. These small, mainly self-selected groups provided a stimulus and workspace for staff, former patients, family members/carers and volunteers to engage with and commit to planning and actioning changes to increase activity opportunities and drive new working practices. However, in the early months of the study it was difficult for staff not involved in co-design groups to envisage whether or how increasing patient activity may affect their roles and working practice. Coherence and cognitive participation, linked to commitment to change and comprehension of the possible benefits of changes in individual and collective practice, developed at different rates across wider staff groups at all sites.

Early 'wins' such as a new 'Social Corner' at site 1 raised awareness amongst all staff, inpatients and visiting family/carers that change was happening. In terms of collective action, some staff incorporated the new space in therapeutic activity or prompted its use independently or with family. Similarly,

For peer review only - http://bmjopen.bmj.com/site/about/guidelines.xhtml

changing a room at site 4 from a wheelchair storage area to a day-room was visible evidence of change and led to its routine use for independent and supervised activity. Such 'public' examples enabled wider understanding of how EBCD/AEBCD led to implementation of environmental and practice changes; this stimulated additional discussion among some staff groups about ways to incorporate increasing activity into day-to-day practice:

"There's been staff involved from physiotherapy and occupational therapy, we meet in a morning and we've handed over what progressions have been from the CREATE study. So say for example we're telling them about the updating of the garden and if we need to take anybody to the garden" (Co-design group member, site 4 post).

Communication and engagement across nursing teams working early, late, on weekends, and night duty was more difficult to achieve; observations indicated project information and changes made were not routinely included in nursing handovers at any site; this initially limited coherence and cognitive participation among nursing staff.

Collective action: challenges in implementing co-designed improvements

One challenge of EBCD/AEBCD is the relatively long time period over which change is planned, implemented and evaluated. Managers largely encouraged staff to participate in EBCD/AEBCD but did not allocate time for participation; staff were encouraged to 'work flexibly'. For nursing staff and HCSWs, attending co-design meetings during shifts was problematic. In site 1 staff sickness and workload challenges meant that three of four EBCD trained staff did not participate in co-design work. At site 4 two AEBCD trained staff, both nurses, attended the joint-meeting but did not participate directly in co-design groups thereafter. Staff led co-design groups in all sites experienced similar challenges but other staff volunteered to take responsibility. Enthusiasm for the project across all sites meant some staff attended joint-meetings on their days off and in sites 1-3, some staff participated in co-design meetings on days off or before shifts. Most staff in co-design groups reported completing activities in their own time. Where implementation did not require substantial change in roles and was perceived as enhancing patients' experience consistent with rehabilitation, staff indicated the extra effort was worthwhile.

"I think it's given me a massive workload, I think it's doubled it...to be fair. But, I was committed, I mean I took it on, but I've enjoyed that, I'm glad for the changes. [.....]I knew we needed changes, so I was happy to help bring the changes". (Staff, site 4, post).

As changes became visible to wider staff groups and involved patients more regularly, staff appeared more receptive to involving external partners. Examples include complementary therapy from a local

For peer review only - http://bmjopen.bmj.com/site/about/guidelines.xhtml

health network (site 1), fortnightly singing with local University students (site 2) and musicians from a community arts group (site 3). Changes elicited positive and negative responses. At site 2 some staff complained the 'Something About Me' (patient information) board mounted behind patients' beds appeared without consultation. However, these staff also said they liked what the board was designed to do. Such comments highlight how difficult it is to secure cognitive participation and commitment to collective action in staff who have limited engagement with implementation of co-designed interventions. Despite some of the challenges outlined, as activity opportunities became more evident shifts in staff perceptions and behaviours suggested collective action and reflexive monitoring were becoming established in most units. Overall team members' perceived changes and acknowledged the positive impact on patients' independence, completion of personal care tasks or engagement in therapy.

Cognitive participation and collective action: influence of managerial authority

Managerial support for service improvements generated through EBCD/AEBCD[36] is important. In sites 1 and 2 researchers set up oversight groups including senior managers, matrons/senior nurses and staff with cross-organisational roles. The commitments of these managers meant interaction was largely through email updates or one-to-one meetings. Oversight groups could not be established at sites 3 or 4 despite invitations to attend AEBCD activity. Oversight group members in sites 1 and 2 supported project activity, helped navigate complex NHS Trust organisational structures and, in specific situations, provided resources to enable co-design group ideas to progress. Having identified senior managerial contacts ensured unit-based staff could activate these lines of support and translate ideas into collective action. This was more difficult in sites 3 and 4 where only the Chief Executive and a therapy lead engaged with AEBCD groups. Site 3 staff completed a sponsored run and the Chief Executive matched the sum raised. Funds (£8000) were used to redesign a day room previously only used by staff and partly to install a kitchenette for patient and family member/carer use.

In all sites, staff working outside of stroke units, including therapy service managers, matrons, patient experience managers, patient safety officers, volunteer co-ordinator and estates managers joined co-design groups. Although not involved in every meeting their participation was often significant in terms of cognitive participation and collective action. NHS staff are acutely aware of resource constraints affecting their services, and most have experienced frustration at organisational barriers to improving services. Barriers identified included infection control and patient safety requirements, delays or inaction when estates work is requested and bureaucratic processes associated with

For peer review only - http://bmjopen.bmj.com/site/about/guidelines.xhtml

including volunteers in unit-based activity. In early co-design meetings at all sites, staff that were otherwise dynamic and enthusiastic advocates for increasing patient activity often expressed the view that such barriers were fixed and would limit what could be achieved. The perception that 'the NHS Trust would not allow' painting murals, adding shelving in patient bays, adding hot drinks facilities for patients and carers to use independently or having volunteers to support patients with social eating, was pervasive. In most sites these perceptions proved to be largely inaccurate; wider hospital staff, patient and volunteer services typically explained how changes could be realised, and importantly, provided examples of where such changes were already operating in the same hospital.

Coherence, cognitive participant and collective action: Facilitating EBCD/AEBCD:

Researchers organised staff, patient and joint events in consultation with EBCD/AEBCD trained staff, recruited former stroke patients and carers and ensured they could attend co-design groups. They booked accessible meeting rooms, arranged reimbursement for patients' and carers' expenses or arranged transport to and from meetings; without this level of support key EBCD/AEBCD events may not have occurred. Researchers co-facilitated staff and joint-meetings; although often initiated by researchers, the joint approach built confidence in EBCD/AEBCD trained staff. In sites 1 and 2 this increased engagement of key staff in EBCD activities. For sites 3 and 4, AEBCD trained staff had less opportunity to work with researchers prior to joint-meetings. In site 3 this appeared to have little impact on co-design meetings, whereas in site 4 team members were less confident in leading co-design meetings. At all sites newsletters were produced by researchers or core team member to share work of co-design groups to wider staff and patient groups (Supplementary file 3).

Collective action and reflexive monitoring: implementing and embedding co-designed changes into stroke unit practice

It took 3-4 co-design groups for staff to recognise potential for changes in spaces for activity and to facilitate supervised and independent activity. For most therapists and therapy assitants (TAs) increasing activity was conceptualised not only as reducing boredom and occupying patients' time but as a therapeutic opportunity. Social activity focused on lunch and breakfast groups at sites 2 and 4, provided opportunities to work on therapy goals including cognitive challenge and functional task practice. These were consistent with therapists' aims in rehabilitation, did not require working in new or different ways and were embraced. In some sites staff reported changes such as the 'Something About Me board' (site 2) or the 'home in the ward' personalisation of bed spaces (sites 1 and 3) made therapy more relevant as staff could draw on information made available to them through these methods. Over time, the work of implementation became more focused on embedding regularly

For peer review only - http://bmjopen.bmj.com/site/about/guidelines.xhtml

occurring group or individually focused activity into therapists and TAs work although the rate at which this occurred varied across sites.

However, post EBCD/AEBCD observations and interviews suggested limited interdisciplinary consideration of activity promotion. Observational data indicated that initially many nurses did not develop an individual or collective understanding of EBCD/AEBCD as something they needed to participate in. This suggested that some nurses team members did not engage with the view that increasing participation in social, cognitive and physical activity was consistent with their view of what constitutes nursing work in stroke units. However, nursing staff overall expressed support for the intentions of the co-design groups but the challenges involved in nurses (in all sites) being able to attend these groups are likely to have impacted on the nurses' collective understanding and engagement in the EBCD/AEBCD process. Ward managers were aware of the challenges Registered nurses (RNs) faced in taking time out of nursing care provision during 8 or 12 hour shifts and allocated time for RNs to attend and participate in co-design meetings. RN and HCSW attendance occurred at site 2 but not in other sites. Observations highlighted nursing staff in each site had high workloads and were frequently affected by staff shortages. Overall, there was limited evidence of RN's encouraging patient focused social or cognitive activity. There were exceptions, two RNs and a HCSW at site 2 actively engaged in co-design work and, toward the end of AEBCD activity, nurses at site 4 routinely worked with therapists in daily breakfast groups. It is possible that as therapist and family led activity increases in these units greater interdisciplinary participation will follow.

Discussion

The CREATE study process evaluation findings confirmed EBCD/AEBCD[29,31,36-36] was feasible to use in stroke units and that this approach facilitated development and implementation of increased activity opportunities. There were four main factors influencing engagement in EBCD/ABCD. Firstly, the participatory approach ensured groups of staff in each unit worked directly with former patients and family/carers over a sustained period to jointly address issues key to increasing social, cognitive and physical activity levels post-stroke. Secondly, the structured, facilitated and time limited nature of EBCD/AEBCD enabled each service to prioritise and agree locally appropriate changes. Thirdly, at the outset of the study photographic evidence gathered by researchers highlighted the typically cold, dark and clinical appearance of the stroke units. Linked to this, participants in all sites chose to work on changing physical environments before focusing on specific types of post-stroke activity. Lastly, EBCD/AEBCD made patient and family/carer experiences real in ways brief questionnaires cannot. Trigger films[29,31] confronted staff, often for the first time in their careers, with the fears, frustrations,

For peer review only - http://bmjopen.bmj.com/site/about/guidelines.xhtml

and positive and negative reality of stroke survivors and family/carers. This proved catalytic in securing staff commitment to bring about change. Trigger films proved powerful in raising staff awareness of ways services may need to change but require skilled facilitation to ensure staff and stroke survivors consider the isues raised in a positive and productive way. In all units, those directly involved in EBCD/AEBCD were positive about the experience and the perceived changes underway in the units. These findings are consistent with those identified in a rapid review of outcomes of co-production studies in acute healthcare settings[40]. The shared sense of purpose and experience developed in co-design groups was consistent with what is termed a 'community of practice'[48] and led to development and implementation of innovative solutions to problems identified by patients and family/carers. Staff more peripheral to EBCD/AEBCD, particularly the larger nursing groups, reported some lack of understanding of and scepticism about the process early in the study. Organising co-design meetings and EBCD/AEBCD updates late morning or late afternoon when nursing staff were often less busy may have facilitated greater involvement and engagement. In addition, securing direct involvement of nurse ward managers in EBCD/AEBCD events was an essential element of securing high level nursing support for change and in sustaining involvement of nursing staff in supporting change. Towards the end of EBCD/AEBCD processes, in interview and during observations most staff indicated positive views on the implementation and embedding of co-designed environmental and practice changes in sites.

Drawing on NPT's mechanisms as a sensitising device in data collection and analysis[44-45] focused attention on individual, group and organisational factors facilitating the use of EBCD/AEBCD to develop and support the implementation and potentially the longer-term sustainability of changes introduced. These included engagement of EBCD/AEBCD trained staff with the skills, expertise and resources of key staff from the wider hospital, including patient experience managers, volunteer coordinators and estates managers. Providing regular project information and updates for managers throughout the EBCD/AEBCD process was instrumental in maintaining their support. Both activities were instrumental in stroke unit staff progressing from understanding the potential of co-producing change with stroke survivors and family carers (coherence and cognitive participation) through committing personally and as staff groups to the kinds of shifts in work patterns needed to facilitate increased patient activity opportunities. These activities together with managerial support were linked to stimulating and supporting collective action in sites.

An additional facilitator supporting implementation of improvements in these sites was engagement with local communities. This occurred at different times and at different levels across the units; sometimes driven by family members, sometimes by patient experience managers or volunteer co-

For peer review only - http://bmjopen.bmj.com/site/about/guidelines.xhtml

ordinators with links to colleges, charities or voluntary organisation. In each unit, there were tangible benefits from community engagement and community groups welcomed the opportunity to provide support to their local hospital. The early evidence suggested this engagement supported collective action and reflexive monitoring with positive feedback from inpatients and family members helping sustain staff groups' commitment to embedding improvements in routine stroke unit practice.

Our findings concur with previous EBCD/AEBCD studies in highlighting the central role of site-based facilitators in establishing and maintaining the EBCD/AEBCD process which in this study were integral to developing and implementing increased activity opportunities[37,40,49-50]. Different models are reported but in the CREATE study researchers undertook this role at each site; they spent less time in sites than reported in other studies, and undertook facilitation alongside data collection. Given the high workload demands and clinical priorities for all stroke unit staff in the study sites, it is unlikely that the EBCD/AEBCD process would have progressed without researcher facilitation. In our view, health services using EBCD/AEBCD need to build in funded facilitator roles in each site. Drawing on NPT highlighted that implementation, in this case of both the EBCD/AEBCD process and the implementation of improvements resulting from the process was not a simple linear process and progressed at different rates in each site.

Barriers to EBCD/AEBCD implementation in all sites were broadly similar to those reported in relation to implementing complex interventions in health services [51-53]. These included staff and local organisational changes at three of the four sites. Less than optimum staffing levels combined with the high level of dependency of stroke survivors in these units made it challenging to free staff to participate in larger EBCD/AEBCD events including joint meetings and celebratory events. Similarly, although fewer staff were required to participate in co-design meetings the frequency, duration and additional workload generated by these meetings proved challenging for EBCD/AEBCD trained and other staff to integrate into their working day. These factors, impacted in particular, on coherence and cognitive participation; understanding the process and potential of the participatory change approach and commiting to that at a personal and practice level often developed slowly in staff not directly involved in co-desing groups. In addition, EBCD or AEBCD approaches were underway for nine and six months respectively, and engaging all stroke unit staff in developing and implementing improvements proved difficult at times. Nonetheless, staff did not resist or sabotage the EBCD/AEBCD process or subsequent changes. The key factor in increasing cognitive participation and progression to collective action was the increasing visibility of changes agreed in co-design meetings. As activity opportunities were integrated into staffs daily or weekly routines, independent and family/carer or volunteer supported patient activity also increased in newly created spaces on the stroke units. At the end of the study these changes appeared to be impacting on unit cultures and staff practice, in

For peer review only

particular more group activities were occurring and were increasingly part of the thinking of staff, particularly therapists, but also with some nursing involvement in each unit.

This process evaluation differed from some other complex intervention evaluations in two ways. Firstly, the use of logic models to define an intervention, its anticipated mechanisms of action and to frame research questions and methods in process evaluation is commonly advocated[42]. However, given the defined approach, stages and structured activities and mechanisms of action identified in previous EBCD/AEBCD studies, a logic model was not developed in the CREATE process evaluation. Secondly, the researchers conducting the process evaluation were members of the core research team rather than working independently of that team. This ensured that researchers were able to both participate in and observe EBCD/AEBCD activities. We acknowledge the limitation that in working closely with staff, former patients and family/carers, researchers were themselves part of the EBCD/AEBCD process and the design and implementation of changes they were evaluating. Recruitment of stroke survivors and family carers to participation in EBCD/AEBCD activities was good across all sites but it proved more difficult to recruit former inpatient stroke survivors to participate in post EBCD/AEBCD evaluation interviews. So, whilst the process evaluation was a comprensive study, we acknowledge that the four participating sites may not be representative of stroke units elsewhere in the UK or other countries. Lastly, the process evaluation was not designed to evaluate the longer-term sustainability of interventions developed in the CREATE study; future studies would benefit from such evaluation.

Conclusion

The findings from the CREATE study, the first of its kind in stroke services, suggest using a co-production approach was instrumental in creating conditions for locally determined former patient and family/carer led change and innovation in service provision. It was possible to utilise EBCD/AEBCD in stroke units providing post-acute and rehabilitation care; this facilitated development and implementation of environmental changes and revisions to work routines which supported increased activity opportunities. NPT's mechanisms were instrumental in identifying facilitators and barriers at the individual, group and organisational levels and attending to these will benefit future implementations of similar approaches. The introduction of EBCD/AEBCD as part of a funded research programme legitimised and supported the co-production activity in these units. However, with appropriate facilitation, managerial engagement and use of wider hospital resources, the approach could be utilised in other rehabilitation stroke services and similar post-acute inpatient environments.

For peer review only - http://bmjopen.bmj.com/site/about/guidelines.xhtml

Acknowledgements:
The authors acknowledge the contribution of the stroke survivors, family members, stroke unit staff, volunteers, hospital managers and local community groups and the support for the study of the four participating NHS Foundation Trust hospitals.

We also wish to acknowledge colleagues who supported elements of the CREATE study, these include Dr Tino Kulnik who conducted pre-implementation interviews for site 1. Dr Alessia Costa (AC), supported ethnographic fieldwork in site 1. Carole Pound, supported co-design events in site 1.

Funding statement: This study was funded by the National Institute for Health Research (NIHR) [under its Health Services and Delivery Research (HS&DR) Programme. Grant Reference Number: 13-11/495]. The views expressed are those of the author(s) and not necessarily those of the NIHR or the Department of Health and Social Care.

Competing interests statement: GR teaches on the EBCD training courses run by 'The Point of Care Foundation' in London. GR is one of the originators of EBCD. The remaining authors declare no other competing interests.

Data sharing statement: No additional data are available.

Author contributions: The CREATE study and embedded process evaluation were designed by FJ, DJC, GR, CMc, RH, GCC and AMc. FJ, DJC, SH and KGW collected and analysed the data. AMc, CMc and RH participated in some joint meetings and celebration events as part of EBCD/AEBCD cycles. DJC developed the manuscript, FJ, GR, CMc, AMc, RH, GCC, SH and KGW reviewed and edited the manuscript. All authors read and approved the final manuscript.

References

For peer review only - http://bmjopen.bmj.com/site/about/guidelines.xhtml

1. Feigin VL, Forouzanfar MH, Krishnamurthi R, et al, on behalf of the Global Burden of Diseases, Injuries, and Risk Factors Study 2010 (GBD 2010) andthe GBD Stroke Experts Group. Global and regional burden of stroke during1990–2010: findings from the Global Burden of Diseases Study 2010. Lancet. 2014; 383(9913):245-255.
2. Stroke Association, UK. State of the Nation. Stroke Statistics. Online. Available at: https://www.stroke.org.uk/sites/default/files/state_of_the_nation_2018.pdf Accessed 10.02.2020
3. Stroke Unit Trialists' Collaboration. Organised inpatient (stroke unit) care for stroke. Cochrane Database of Systematic Reviews 2013, Issue 9. Art. No.: CD000197. DOI: 10.1002/14651858.CD000197.pub3.
4. Keith RA. Activity patterns of stroke rehabilitation unit. Soc Sci Med.1980; 14:575-580.
5. Keith, R.A. Cowell,K.S., Time use of stroke patinets in three rehabilitation hospitals. Social Science and Medicine. 1987. 24,6:529-533.
6. Lincoln NB, Gamlen R, Thomason H. Behavioural mapping of patients on a stroke unit. Int Disabil Stud.1989; 11:149-154.
7. Bernhardt J, Dewey H, Thrift A & Donnan G. Inactive and alone: physical activity within the first 14 days of acute stroke unit care. Stroke 2004:35;1005-9.
8. Janssen H, Ada L, Bernhardt J, McElduff P, Pollack M, Nilsson M, Spratt NJ (2014). An enriched environment increases activity in stroke patients undergoing rehabilitation in a mixed rehabilitation unit: a pilot non-randomized controlled trial. Disabil Rehabil. 36: 255-262.
9. Prakash, V., Shah, M. A., and Hariohm, K. (2016). Family's presence associated with increased physical activity in patients with acute stroke: an observational study. *Braz. J. Phys. Ther.* 20, 306–311. doi: 10.1590/bjpt-rbf.2014.0172
10. Rosbergen, I. C. M., Grimley, R. S., Hayward, K. S., Walker, K. C., Rowley, D., Campbell, A. M., et al. (2017). Embedding an enriched environment in an acute stroke unit increases activity in people with stroke: a controlled before-after pilot study. *Clin. Rehabil.* 31, 1516–1528. doi: 10.1177/0269215517705181
11. Foley N, McClure JA, Meyer M, Salter K, Bureau Y, Teasell R. Inpatient rehabilitation following stroke: amount of therapy received and associations with functional recovery. Disabil Rehabil 2012; 34(25): 2132-8.
12. Otterman NM, van der Wees PJ, Bernhardt J, Kwakkel G. Physical therapists' guideline adherence on early mobilization and intensity of practice at Dutch acute stroke units a country-wide survey. Stroke 2012; 43(9): 2395-401.
13. Merchant, J., Kitsos,G., Ashby,S., Kitsos,A., Hubbard, I. Occupational therapy and physiotherapy in acute stroke: do rural patients receive less therapy? Stroke Research and Treatment. 2016. http:/dx.doi.org/10.1155/2016/1582706.
14. Clarke,D,J.; Burton,L.J.; Tyson,S., Rodgers,H., Drummond,A., Palmer,., Hoffman,A., PrescottM., Tyrrell, P., Brkic, L., Grenfell,K., Forster,A. Why do stroke survivors not receive recommended amounts of active therapy? Findings from the ReAcT study, a mixed-methods case-study evaluation in eight stroke units. Clinical Rehabilitation 1–14. 2018 DOI: 10.1177/0269215518765329
15. Fini NA, Holland AE, Keating J, Simek J, Bernhardt,J. How Physically Active Are People Following Stroke? Systematic Review and Quantitative Synthesis. Phys Ther. 2017 Jul 1; 97(7):707-717.
16. Luker J, Murray C, Lynch E, Bernhardsson S, Shannon M, Bernhardt J. Carers' Experiences, Needs, and Preferences During Inpatient Stroke Rehabilitation: A Systematic Review of Qualitative Studies. Arch Phys Med Rehabil. 2017;98(9):1852–62 e1813.
17. Ellul, J., Watkins, C., Ferguson, N., Barer, D., and Rowe,J. "Increasing patient engagement in rehabilitation activities". Clinical Rehabilitation. 1993. 7(4):297-302.
18. Newall, J.T., Wood, V.A., Hewer. R,L., and Tinson, D,J. (1997). "Development of a neurological rehabilitation environment: an observational study". Clinical Rehabilitation. 11(2): 146-55.
19. Scrivener K, Sherrington C, Schurr K. Exercise dose and mobility outcome in a comprehensive stroke unit: description and prediction from a prospective cohort study. J Rehabil Med 2012;44:824–9.

For peer review only

20. Kwakkel G, van Peppen R, Wagenaar RC, et al. Effects of augmented exercise therapy time after stroke: a meta-analysis. Stroke 2004;35:2529–36.

21. Sarkamo T, Tervaniemi M, Laitinen S, et al. Music listening enhances cognitive recovery and mood after middle cerebral artery stroke. Brain 2008;131:866–76.

22. Rosbergen, I. C., Grimley, R. S., Hayward, K. S., Walker, K. C., Rowley, D., Campbell, A. M., McGufficke, S., Robertson, S. T., Trinder, J., Janssen, H., & Brauer, S. G. (2016). The effect of an enriched environment on activity levels in people with stroke in an acute stroke unit: protocol for a before-after pilot study. *Pilot and feasibility studies*, *2*, 36. https://doi.org/10.1186/s40814-016-0081-z

23. Khan,F. Amatya,B., Elmalik,A, Lowe,M., Ng,L., Reid,I., Galea,M.P. et al, An Enriched Environmental Programme During Inpatient Neuro-rehabilitation: A Randomized Controlled Trial. Journal of Rehabilitation Medicine, 48, (5): 417-425(9)2016;

24. Rosbergen, I. C., Grimley, R. S., Hayward, K. S., & Brauer, S. G. (2019). The impact of environmental enrichment in an acute stroke unit on how and when patients undertake activities. Clinical Rehabilitation, 33(4), 784–795. https://doi.org/10.1177/0269215518820087

25. McDonald, M. W., Hayward, K. S., Rosbergen, I., Jeffers, M. S., & Corbett, D. (2018). Is Environmental Enrichment Ready for Clinical Application in Human Post-stroke Rehabilitation?. Frontiers in behavioral neuroscience, 12, 135. doi:10.3389/fnbeh.2018.00135.

26. Anåker, A., von Koch, L., Sjöstrand, C., Bernhardt, J., & Elf, M. (2017). A comparative study of patients' activities and interactions in a stroke unit before and after reconstruction-The significance of the built environment. PloS one, 12(7), e0177477. https://doi.org/10.1371/journal.pone.0177477

27. Shannon,M.M., Elf,M., Churilov,L., Olver,J., Pert,A., Bernhardt,J. Can the physical environment itself influence neurological patient activity?, Disability and Rehabilitation, 2019. 41:10, 1177-1189, DOI: 10.1080/09638288.2017.1423520

28. Needham, C. and Carr, S. Co-production: an emerging evidence base for adult social care transformation. Research Briefing 31. 2009. Social Care Institute for Excellence.

29. Robert G , Cornwell J , Locock L , et al . Patients and staff as co-designers of healthcare services. BMJ 2015;350:g7714.

30. Batalden P. Getting more health from healthcare: quality improvement must acknowledge patient coproduction—an essay by Paul Batalden. BMJ. 2018 Sep 6;362:k3617.

31. Point of Care Foundation, Experience-based co-design toolkit, Available from:https://www.pointofcarefoundation.org.uk/resource/experience-based-co-design-ebcd-toolkit/ Accessed 10/02/2020.

32. Coldham,T., Denegri, S., Green,G. Staniszewska,S., Tembo,D., Torok,K. and Turner,K. 2018. Guidance on co-producing a research project. Southampton, INVOLVE. https://www.invo.org.uk/posttypepublication/guidance-on-co-producing-a-research-project/ Accessed 10/02/2020.

33. Boaden, R. Push, pull or co-produce? Journal of Health Services Research and Policy.2020. 25 (2): 67-69

34. Co-production of health and wellbeing Scotland. Online: http://www.coproductionscotland.org.uk/about/what-is-co-production/ Accessed 10/02/2020

35. Bate SP , Robert G . Bringing user experience to health care improvement: the concepts, methods and practices of experience-based design. Oxford: Radcliffe Publishing, 2007.

36. Donetto,S., Tsianakas,V, Robert,G. Using Experience-based Co-design (EBCD) to improve the quality of healthcare: mapping where we are now and establishing future directions. National Nursing Research Unit. Final Report. Kings College London. 2014.

37. Locock L, Robert G, Boaz A, Vougioukalou S, Shuldham C, Fielden J, *et al.* Testing accelerated experience-based co-design: a qualitative study of using a national archive of patient experience narrative interviews to promote rapid patient-centred service improvement. *Health Serv Deliv Res* 2014;2(4)

For peer review only - http://bmjopen.bmj.com/site/about/guidelines.xhtml

38. Jones F, Gombert-Waldron K, Honey S, Cloud G, Harris R, Macdonald A, et al. Using co-production to increase activity in acute stroke units: the CREATE mixed-methods study. Health Serv Deliv Res 2020;8(35) https://www.journalslibrary.nihr.ac.uk/hsdr/hsdr08350#/full-report.
39. Jones, F. Gombert,K., Honey,S. Cloud,G, Harris, R., Macdonald,A., McKevitt,C., Robert,G., Clarke,D.J. Addressing inactivity after stroke: The Collaborative Rehabilitation in Acute Stroke (CREATE) study. International Journal of Stroke. 2020. DOI: 10.1177/174793020969367
40. Clarke D, Jones F, Harris R on behalf of the Collaborative Rehabilitation Environments in Acute Stroke (CREATE) team, et al What outcomes are associated with developing and implementing co-produced interventions in acute healthcare settings? A rapid evidence synthesis BMJ Open 2017;7:e014650. doi: 10.1136/bmjopen-2016-014650
41. Craig P, Dieppe P, Macintyre S, et al. Developing and evaluating complex interventions: new guidance: MRC, 2008.
42. Moore GF, Audrey S, Barker M, Bond L, Bonell C, Hardeman W, Moore L, O'Cathain A, Tinati T, Wight D, Baird J. Process evaluation of complex interventions: Medical Research Council guidance. BMJ 2015; 350: h1258.
43. Royal College of Physicians. SSNAP Biannual national Acute Stroke Organisational audit data, reported in 2016. London, Royal College of Physicians.
44. May, C., Rapley, T., Mair, F.S., Treweek, S., Murray, E., Ballini, L., Macfarlane, A. Girling, M. and Finch, T.L. (2015) Normalization Process Theory On-line Users' Manual, Toolkit and NoMAD instrument. Available from http://www.normalizationprocess.org Accessed 10/02/2020.
45. May,C., Cummings, A., Girling,M., Bracher,M., Mair, F.S., May,C.M., Murray, E., Myall, M., Rapley, T., Finch, T. Using Normalisation Process Theory in feasibility studies and process evaluations of compelx healthcare interventions: a systematic review. Implementation Science. 2018. 13:80, DOI: 10.1186/s13012-018-0758-1
46. NVivo 10 QSR International PTY 2015
47. Braun,V., Clarke,V. Using thematic analysis in psychology, Qualitative Research in Psychology,2006. 3:2, 77-101, DOI: 10.1191/1478088706qp063oa 2006
48. Wenger, E. 1998 Communities of Practice: learning, meaning, and identity. Cambridge University Press
49. Tollyfield, R. Facilitating an accelerated experience-based co-design project. *Brit Journal of Nurs*, 2014, 23, (3):136-141
50. Larkin, M Boden, ZVR, Newton, E et al, On the Brink of Genuinely Collaborative Care: Experience-Based Co-Design in Mental Health. *Qualitative Health Research* 2015, 25(11) 1463–1476

51. Grol R, Grimshaw J. From best evidence to best practice: effective implementation of change in patients' care. Lancet. 2003;362:1225–1230.

52. Grimshaw JM, Eccles MP, Lavis JNet al. . Knowledge translation of research findings. Implement Sci. 2012;7:50.
53. Child, S., Goodwin, V., Garside, R. et al. Implementation Sci (2012) 7: 91. https://doi.org/10.1186/1748-5908-7-91

For peer review only - http://bmjopen.bmj.com/site/about/guidelines.xhtml

For peer review only

For peer review only - http://bmjopen.bmj.com/site/about/guidelines.xhtml

Table 1 Unit characteristics

	Site 1 (EBCD)	Site 2 (EBCD)	Site 3 (AEBCD)	Site 4 (AEBCD)
**Number of stroke beds**	24	24	26	26 beds across two adjoining wards (14 and 12 beds)
**Hospital type**	District general hospital with 629 beds	District general hospital with 500 beds	District general hospital with 700 beds	District general hospital with 600 beds
**Number of stroke patients treated per year**	195	978*	250	640*
**Typical length of stay (days)**	28	13*	28	21*
**7-day therapy service**	No	An OT and PT worked on Saturday covering the acute and rehabilitation units. A Stroke Rehabilitation Assistant	No	No

For peer review only - http://bmjopen.bmj.com/site/about/guidelines.xhtml

		worked on the rehabilitation unit on Saturdays and Sundays.		
**Performance in National Acute Organisational Audit (RCP, 2017).**	Achieved 7 of the 10 key indicators.	Achieved 4 of 10 key indicators.	Achieved 8 of the 10 key indicators.	Achieved 5 of the 10 key indicators.

* Data for Sites 2 and 4 include data for Hyperacute/Acute units in the same hospital.

Table 2 Number of participants by data generation method

**Site**	**Staff interviews**	**Patient interviews**	**Carer interviews**	**Non-participant Observations (hours)**
**Site 1 pre EBCD**	N=13	N= 9	N= 4	50
**Site 1 post EBCD**	8	5	5	47
**Site 2 pre EBCD**	15	9	4	48

For peer review only - http://bmjopen.bmj.com/site/about/guidelines.xhtml

**Site 2 post EBCD**	7	6	2	44
**Site 3 pre AEBCD**	6	9	3	50
**Site 3 post AEBCD**	8	6	3	37
**Site 4 pre AEBCD**	7	4	3	44
**Site 4 post AEBCD**	12	5	3	46
**TOTAL**	**76**	**53**	**27**	**366 hours**

For peer review only - http://bmjopen.bmj.com/site/about/guidelines.xhtml

Table **3 Normalisation Process Theory**

NPT constructs	Components	Explanation
**Coherence**	• Differentiation<br>• Communal specification<br>• Individual specification<br>• Internalisation	The sense making work that people do individually and collectively when faced with implementing changes to existing working practices. This would include differentiating new practices from existing work and thinking through not only the perceived value and benefits of desired/planned changes but also what work will be required of individual people in a setting to bring about these changes.
**Cognitive Participation**	• Initiation<br>• Enrolment<br>• Legitimation<br>• Activation	The work that people need to do to engage with and commit to a new set of working practices. This often requires bringing together those who believe in and are committed to making changes happen. This also involves people working together to define ways to implement and sustain the new working practices.
**Collective Action**	• Interactional workability<br>• Relational integration<br>• Skill set workability<br>• Contextual integration	The work that will be required of people to actually implement changes in practices, including preparation and/or training of staff. Often this entails rethinking how far existing work practices and the division of labour in a setting will have to be changed or adapted to implement the new practices. This requires consideration of not only who will do the work required, but also the skills and knowledge of people who will do the work and the availability of the resources they need to enact and sustain the new working practices.
**Reflexive monitoring**	• Systematisation<br>• Communal appraisal<br>• Individual appraisal<br>• Reconfiguration	Peoples' individual and collective on-going informal and formal appraisal of the usefulness or effectiveness of changes in working practices. This involves considering how the new practices affect the other work required of individuals and groups and whether the intended benefits of the new working practices are evident for the intended recipients and staff.

For peer review only - http://bmjopen.bmj.com/site/about/guidelines.xhtml

Table 4: **NPT mechanisms and components identified during the EBCD/AEBCD process**

Observed processes	Combinations of NPT mechanisms and components identified during the EBCD/AEBCD process	Facilitators influencing use of EBCD/AEBCD as a process to develop and implement improvements to increase activity opportunity	Barriers and challenges to using EBCD/AEBCD as a process to develop and implement improvements to increase activity opportunity
Staff, inpatients, former and carers made sense of and engaged with EBCD/AEBCD at different rates over time.	*Coherence:*<br>• *Differentiation*<br>• *Individual specification*<br><br>*Cognitive participation:*<br>• *Initiation*<br>• *Enrolment*	• Small groups of staff with access to EBCD/AEBCD team training;<br>• Researcher presence during observation and interview periods provided; opportunities to explain EBCD/AEBCD to more staff one to one and in small groups;<br>• Patients, former patients and carers became aware of the study through observations and interview participation.	• Without access to EBCD/AEBCD training -challenging in all sites to facilitate individual and collective understanding and engagement in wider stroke unit and hospital staff and volunteers;<br>• Delay between training and commencing EBCD/AEBCD activity in all sites- early interest in the study dissipated- impacted on understanding what EBCD/AEBCD process could achieve and would mean for wider staff groups;<br>• Inpatients did not participate in co-design meetings and had no direct in engagement creating increased activity opportunities.
The facilitated EBCD/AEBCD process was perceived to be different and more effective than prior change initiatives designed to improve activity opportunities.	*Coherence:*<br>• *Differentiation*<br>• *Individual specification*<br>• *Communal specification*<br><br>*Cognitive participation:*<br>• *Initiation*<br>• *Enrolment*<br>• *Legitimation*<br><br>*Collective action:*<br>• *Interactional workability*	• Structured, time limited and externally facilitated approach of EBCD/AEBCD;<br>• Sites 1 and 2: large numbers of staff participate in separate and joint meetings;<br>• Sites 3 and 4 (AEBCD) joint meetings important in engaging staff in the process;<br>• EBCD/ABCD as a tried and tested service improvement model- legitimised staff time and resource allocation.	• Across all sites limited numbers of stroke unit staff, wider hospital staff, volunteers, former patients and carers can participate in EBCD/AEBCD process activities.
Former patient & family member participation in EBCD/AEBCD	*Coherence:*<br>• *Individual specification*<br>• *Communal specification*	• Trigger films demonstrate commonality and difference in experiences and facilitate former patients and carers identification as a group with shared beliefs on changing patients' activity experiences in their local stroke units.	• Recruitment and retention of a representative range of former patients & family members to participate in EBCD/AEBCD was challenging in some sites; numbers participating were higher at both London sites.

For peer review only - http://bmjopen.bmj.com/site/about/guidelines.xhtml

	Cognitive participation:<br>• *Initiation*<br>• *Enrolment*<br>• *Legitimation*<br><br>*Collective action:*<br>• *Interactional workability*	• Trigger films are a powerful means of highlighting stroke survivors' and carers' experiences related to inactivity and activity in stroke units-these heighten staff understanding of stroke survivors' individual and collective experiences<br>• EBCD/AEBCD engages participants as equal partners in the improvement process.	
Small numbers of staff, volunteers, former patients, family members participate in co-design groups.	*Coherence:*<br>• *Communal specification*<br>• *Internalisation*<br><br>*Cognitive participation:*<br>• *Enrolment*<br>• *Legitimation*<br>• *Activation*<br><br>*Collective action:*<br>• *Interactional workability*<br>• *Relational integration*	• Co-design groups were effective with small numbers of regular participants<br>• Co-design groups tended to foster participants' continued commitment to individual and collective action and practice change;<br>• Co-design groups provided a mechanism to involve non stroke unit staff who could suggest and sanction improvements related to estates and community involvement;<br>• Co-design groups were the drivers of change and improvement	• Wider staff groups had less involvement in planning and actioning change planned by co-design groups;<br>• Staff numbers varied with many more nurses and HCSWs working in stroke teams than therapists, stroke SRAs and medical staff;<br>• Strategies for communicating planned change had varied success (newsletters, using routine staff information exchange events, open afternoons);<br>• Staff participants in co-design groups had increased workloads, typically with no additional time allocation.
Researcher facilitation of EBCD/AEBCD process	*Cognitive participation:*<br>• *Initiation*<br>• *Enrolment*<br>• *Legitimation*<br>• *Activation*<br><br>*Collective action:*<br>• *Interactional workability*<br>• *Relational integration*<br>• *Skill set workability*	• Researcher facilitation of the structured EBCD/AEBCD enabled key staff to commit to participation in co-design groups and to developing and implementing improvements whilst maintaining their usual work roles and responsibilities;<br>• Researcher facilitation was a key part of recruitment, retention and participation of former patients and carers;<br>• Researcher facilitation was important in enabling and sustaining involvement of wider hospital staff, volunteers and community involvement;	• Researcher facilitation could mean some staff relied on researchers to lead and progress the EBCD/AEBCD process rather than assuming leadership roles;<br>• Time required to contact, engage with, involve and sustain participation of non stroke unit staff, volunteers and community groups was initially very high;<br>• Evidence in other EBCD projects of improvement activity stalling or ceasing when facilitator role ends.

For peer review only - http://bmjopen.bmj.com/site/about/guidelines.xhtml

		• Researcher facilitation supported key stroke unit staff in assuming responsibility for improvements after the CREATE study ended.	
The influence of managerial authority and support for stroke unit staff participation and improvement activity	*Cognitive participation:*<br>• *Initiation*<br>• *Enrolment*<br>• *Legitimation*<br><br>*Collective action:*<br>• *Interactional workability*<br>• *Relational integration*<br>• *Skill set workability*<br>• *Contextual integration*	• Managerial support for involvement of staff in the EBCD/AEBCD project was critical to the progression of the process in all sites-both in terms of joint meeting attendance for larger groups of staff and those staff routinely involved in co-design groups;<br>• Managerial support and clinical directorate and stroke unit level was required for co-design groups to navigate complex NHS Trust organisational structures and, in leveraging resources to enable co-design group ideas to progress;<br>• Involving managers with experience of service improvement elsewhere in the hospital in co-design groups ensured myths and misconceptions about what changes were possible in the organisation were quickly addressed and corrected.	• Securing direct involvement of non stroke unit managers in a project which did not directly address clinical outcomes or National Clinical Audit targets was challenging in all sites;<br>• Stroke unit staff were reluctant to take direct action involving estates to effect environmental support or use of volunteers on units in the absence of managerial support ay clinical directorate or stroke service level.
Challenges related to the duration of the change process and time taken for improvements to be introduced.	*Cognitive participation:*<br>• *Legitimation*<br>• *Activation*<br><br>*Collective action:*<br>• *Relational integration*<br>• *Skill set workability*<br>• *Contextual integration*<br><br>*Reflexive monitoring:*<br>• *Systematisation*<br>• *Communal appraisal*<br>• *Individual appraisal*	• Full EBCD cycles took approximately 9 months to complete;<br>• AEBCD cycles took approximately 6 months to complete;	• Some seemingly simple improvements took many weeks to plan, develop and implement e.g. improved web access; making iPads accessible and secure; providing new and welcoming signage; facilitating on unit volunteer and community group activities;<br>• Improvements involving changes in space use, environment or volunteer involvement required negotiation with and sometimes additional funding from managers; this could take long periods of time or be blocked or ignored by managers.

For peer review only - http://bmjopen.bmj.com/site/about/guidelines.xhtml

Implementing and embedding co-designed changes into stroke unit practice	*Collective action:*<br>• *Relational integration*<br>• *Skill set workability*<br>• *Contextual integration*<br><br>*Reflexive monitoring:*<br>• *Systematisation*<br>• *Communal appraisal*<br>• *Individual appraisal*	• 'Visible' improvements including new spaces for activity and environmental changes helped staff not involved in co-design groups understand the kinds of improvements occurring and led to inclusion of these spaces or environmental changes in the practice of therapists and nurses;<br>• Introduction and use of co-designed improvements such as the 'Something about me board' and personalising bed spaces with photos and personal objects were reported to prompt more personalised supervised and independent activity opportunities;<br>• Where new activity opportunities supported or could be added to existing rehabilitation practice these quickly became incorporated in the routines of therapists and nurses;<br>• Agreed, routine volunteer led activity was recognised as valuable in increasing social and cognitive activity opportunities for stroke survivors-this reduced demand on stroke unit staff and helped ensure volunteers had a defined role and contribution on the unit.	• Involving large and diverse staff groups as in stroke units in developing and implementing improvements proved challenging;<br>• Some staff felt they were not consulted about improvements which would require their on-going involvement e.g. completion of required information on the 'something about me boards'; preparing inpatients for and supporting them in breakfast club activity;<br>• Ensuring unit wide change , across disciplines, in encouraging and supporting activity opportunities was more difficult than securing uni-disciplinary commitment to increasing activity opportunities;<br>• Routine volunteer activity on units was variably received-staff need to be confident volunteers have received appropriate stroke specific training for example if involved in communication support or mealtime support for stroke survivors.

For peer review only - http://bmjopen.bmj.com/site/about/guidelines.xhtml

Figure 1: CREATE study design and methods with embedded process evaluation

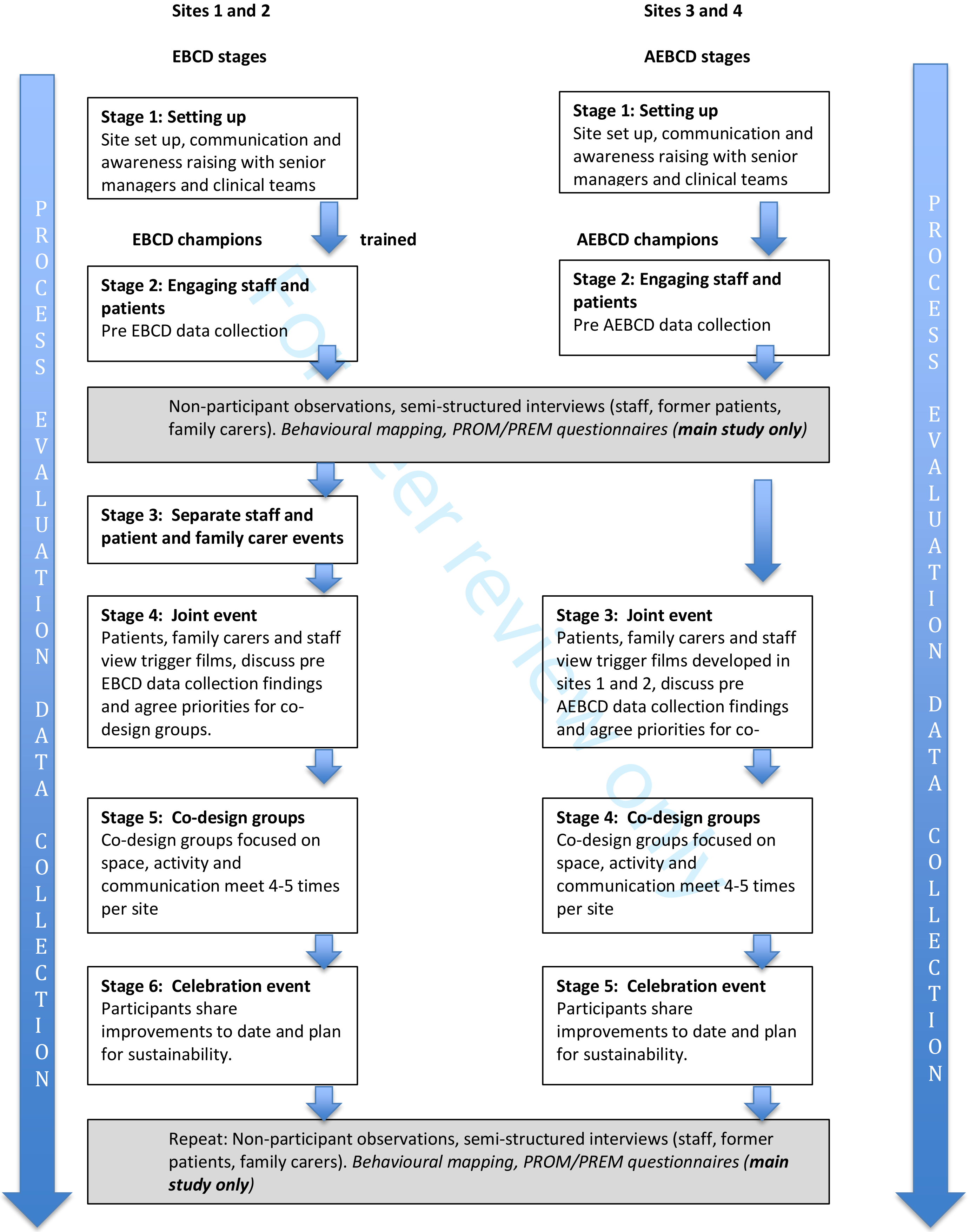

Supplementary File 1: Data collection linked to Normalisation Process Theory

Focus for and use of Normalisation Process Theory in the process evaluation of the CREATE study. NB: the elements are not necessarily progressed through in a linear way; there is some degree of cyclical movement back and forth between elements.			
**NPT element**	**Focus**	**Data collection (keep in NVivo or as word documents)**	**Notes**
**Coherence**	EBCD/AEBCD training for staff from sites	Researcher reflective notes on the training days<br><br>Point of Care Foundation training feedback sheets (anonymised) were available for the full day training and then feedback/post it notes from the half day training.	
	Informal staff comments and observations during: non-participant observations	Researchers' reflective notes on staff perceptions and understanding of the aims of the study (increase patient activity outside of therapy through co-designed interventions.	Include any evidence of access to the Kings Fund Toolkit, any evidence of dialogue between stroke unit teams (e.g. in handovers, at MDT or other staff meetings)
	Staff interviews pre interventions with:<br>a) EBCD/AEBCD champions at each site<br>b) Remaining staff interviewees	Interview transcripts (where these occurred)<br><br>Researchers' reflective notes on staff perceptions and understanding of the aims of the study and any evidence of actions to involve the wider stroke unit team<br>Researchers' reflective notes on staff perceptions and understanding of the aims of the study<br>Review documentary evidence (notes of meetings/action plans/reviews of action plans)	(Overlaps with cognitive participation, collective action and reflexive monitoring)
	Study Oversight Groups (SoG) meetings	SoG members at sites include: Deputy Directorate Manager, Therapy Lead (Manager), Service Improvement Lead, Volunteer Manager, Stroke Specialist Nurse.	These were largely virtual meetings - review notes and email trails/responses from any SoG members

Supplementary File 1: Data collection linked to Normalisation Process Theory

**Cognitive participation**	Staff interviews post interventions with:<br>a) EBCD/AEBCD champions at each site<br>b) Remaining staff interviewees	Interview transcripts<br><br>Researchers' reflective notes on staff perceptions and understanding of the aims of the study and any evidence of actions to involve the wider stroke unit team	***Overlaps with coherence***
	Preparation for and conduct of staff event and patient event and joint event	Patient and staff questionnaire* feedback<br>EBCD champions individual reflective summary (Guide given)<br>Researchers' reflective notes on staff perceptions and understanding of the aims of the study and any evidence of actions to involve the wider stroke unit team	Questionnaires and reflective summary sheets adapted from those used in the Locock et al (2014) study
	Co-design group meetings	Patient and staff questionnaire feedback<br>EBCD champions individual reflective summary<br>Review documentary evidence (notes of meetings/action plans/reviews of action plans)<br>Researchers' reflective notes on staff perceptions and understanding of the co-design work and any evidence of actions to involve the wider stroke unit team.	As above- seek questionnaires and reflective summaries after each event or meeting.
	Study Oversight Groups (SoG) any ongoing meetings	Researchers' reflective notes on staff perceptions and understanding of the progress of the study and of staff and patient involvement in this. Review documentary evidence (notes of meetings/action plans/reviews of action plans)	

For peer review only

For peer review only - http://bmjopen.bmj.com/site/about/guidelines.xhtml

Supplementary File 1: Data collection linked to Normalisation Process Theory

**Collective action**	Co-design groups<br>And implementation activity<br><br>And feedback from the celebratory events at both sites	Any patient and staff questionnaire* feedback<br>EBCD champions individual reflective summary<br>Review documentary evidence (notes of meetings/action plans/reviews of action plans)<br><br>Researchers' reflective notes on staff's and patients' and carers' engagement with the co-design work and evidence of actions to involve the wider stroke unit team. (E.g. be alert to discussion at MDT or other meetings), evidence of impacts on existing work of staff and how this is managed/discussed.<br><br>Researchers' reflective notes on range of interventions/activities introduced and how these are managed by individuals and groups (e.g. therapists/nurses/volunteers) and whether and how these are publicised by the EBCD champions or others	
**Reflexive monitoring**	Co-design groups<br>And implementation activity<br><br>Staff interviews with:<br>a) EBCD/AEBCD champions at each site<br>b) Remaining staff<br><br>Patient and carer interviews<br><br>Informal staff comments and observations during: non-participant observations	Data collection as above<br><br>Interview transcripts<br><br>Researchers' reflective notes on staff perceptions of the study and how the co-design work and interventions have affected patients' activity levels and overall inpatient experience.<br>Review the involvement of the wider stroke unit team in interventions to increase activity levels<br><br>Review documentary evidence (notes of meetings/action plans/reviews of action plans)<br><br>Interview transcripts<br><br>Researchers' reflective notes on staff perceptions and understanding of the outcomes of the study (increase patient activity outside of therapy through co-designed interventions.	**Links back to Cognitive participation**- review reflexive monitoring activity at the when co-design groups are established and as interventions/activities are piloted

Supplementary File 1: Data collection linked to Normalisation Process Theory

**Additional focus on:**	Absence of separate staff and patient meetings in AEBCD sites<br><br>Shortened timescale for co-design at AEBCD sites<br><br>Differences in level of patient and carer recruitment for EBCD/AEBCD groups between sites.	Consider the impact on the joint meeting and progression to co-design work<br><br>Examples are numbers of people involved different sites.	
**Organisational factors impacting on set up of CREATE at sites:**	Consider: managerial involvement or its absence, project disruption at sites interest and support at the wider organisational level or beyond	Consider other organisational factors relevant to the conduct of the study at each site including:<br><br>Key staff absence or key staff leaving the organisation<br><br>Ongoing or specific staffing problems<br><br>Positive/innovative factors such as the fundraising and design competition at site 3.<br><br>And the pre-existing funding for day room refurbishment at Site 2, and the ongoing Rooting and Fruiting gardening project at Site 4	

For peer review only

For peer review only - http://bmjopen.bmj.com/site/about/guidelines.xhtml

Supplementary file 2: Demographic data all participants

Patient demographics

**Site 1 Patient interviews (n= 9) pre-implementation of EBCD**	
**Age**	**Gender**
65 +/-12 years	M = 5<br>F = 4
**Site 1 Patient interviews (n= 5) post-implementation of EBCD**	
65 +/-13 years	M = 3<br>F = 2
**Site 2 Patient interviews (n= 9) pre-implementation of EBCD**	
68 =/- 18 years	M= 5<br>F= 4
**Site 2 Patient interviews (n=6) post-implementation of EBCD**	
73 +/- 12 years	M =6
**Site 3 Patient interviews (n= 9) pre-implementation of EBCD**	
61 +/- 16 years	M = 5<br>F = 4
**Site 3 Patient interviews (n= 6) post-implementation of EBCD**	
67 +/- 18 years	M = 4<br>F = 2
**Site 4 Patient interviews (n= 4) pre-implementation of EBCD**	
72 +/- 7 years	M = 3<br>F = 1
**Site 4 Patient interviews (n= 6) post-implementation of EBCD**	
68 +/- 10 years	M = 2<br>F = 4

F female, M male

Family carer demographics

**Site 1 Carer interviews (n= 4) pre-implementation of EBCD**	
Age	Gender
45-54	F
18-24	M
55-64	M
55-64	F
**Site 1 Carer interviews (n= 5) post-implementation of EBCD**	
18-24	M
55-64	M
45-54	F
65-74	M
75-84	F

For peer review only

Site 2 Carer interviews (n=4) pre-implementation of EBCD	
55-64	F
65-74	F
65-74	F
75-84	M
**Site 2 Carer interviews (n= 2) post-implementation of EBCD**	
75-84	F
75-84	F
**Site 3 Carer interviews (n=3) pre-implementation of AEBCD**	
65-74	F
55-64	F
75-84	F
**Site 3 Carer interviews (n=3) post-implementation of AEBCD**	
65-74	F
65-74	M
18-24	F
**Site 4 Carer interviews (n=2) pre-implementation of AEBCD**	
55-64	F
65-74	M
**Site 4 Carer interviews (n=5) post-implementation of AEBCD**	
85-94	M
65-74	M
55-64	M
65-74	M
55-64	F

Demographic data staff participants

Site 1 Staff interviews (n=13) pre-implementation of EBCD	
**Age**	**Profession**
45-54	Doctor
25-34	RN
35-44	RN
35-44	HCSW
45-54	HCSW
45-54	Domestic
35-44	OT
25-34	OT
45-54	PT
25-34	PT
35-44	Clinical Psychologist
25-34	SALT
35-44	Dietician
**Site 1 Staff interviews (n=8) post-implementation of EBCD**	
25-34	SALT
25-34	PT
35-44	OT
35-44	Dietician
35-44	SRA
35-44	Operations manager
25-34	RN
45-54	Consultant
**Site 2 Staff interviews (n=15) pre-implementation of EBCD**	
35-44	PT
25-34	PT
18-24	SRA
25-34	HCSW

For peer review only

35-44	SALT
35-44	OT
25-34	Dietician
25-34	OT
25-34	RN
25-34	RN
25-34	RN
55-64	Volunteer
25-34	Dietician
35-44	HCSW
35-44	Doctor
**Site 2 staff interviews (n= 7) post-implementation of EBCD**	
25-34	RN
25-34	PT
35-44	HCA
25-34	SRA
25-34	RN
25-34	OT
45-54	HCSW
**Site 3 Staff interviews (n= 6) pre-implementation of AEBCD**	
35-44	Clinical Psychologist
35-44	OT
25-34	PT
35-44	RN
45-54	PT
45-54	Doctor
**Site 3 Staff interviews (n= 8) post-implementation of AEBCD**	
35-44	Clinical Psychologist
25-34	OT
25-34	PT
45-54	RN
45-54	PT
45-54	Doctor
35-44	HCSW
35-44	SRA
**Site 4 Staff interviews (n= 7) pre-implementation of AEBCD**	
35-44	TA
35-44	Doctor
45-54	RN
25-34	PT
25-34	OT
55-64	RN
45-54	SALT
**Site 4 Staff interviews (n= 9) post-implementation of AEBCD**	
25-34	SALT
35-44	RN
25-34	HCSW
25-34	PT
55-64	RN
45-54	RN
25-34	OT
45-54	TA
35-44	RN

PT physiotherapist, OT, occupational therapist, SALT speech and language therapist, RN, registered nurse, SRA stroke rehabilitation assistant, TA therapy assistant, HCSW healthcare support worker.

For peer review only

Supplementary file 3: Number of Co-design groups held

	**Co-design participants**			**Number of co-design group meetings**
	**Stroke survivors**	**Carers**	**Staff (includes volunteers)**	
**Site 1 (EBCD)**	5	6	13	10
**Site 2 (EBCD)**	4	4	12	9
**Site 3 (AEBCD)**	11	7	14	15
**Site 4 (AEBCD)**	3	4	15	9
**TOTAL**	**23**	**21**	**54**	**43**

For peer review only

COREQ (COnsolidated criteria for REporting Qualitative research) Checklist

A checklist of items that should be included in reports of qualitative research. You must report the page number in your manuscript where you consider each of the items listed in this checklist. If you have not included this information, either revise your manuscript accordingly before submitting or note N/A.

Topic	Item No.	Guide Questions/Description	Reported on Page No.
**Domain 1: Research team and reflexivity**			
Personal characteristics			
Interviewer/facilitator	1	Which author/s conducted the interview or focus group?	
Credentials	2	What were the researcher's credentials? E.g. PhD, MD	
Occupation	3	What was their occupation at the time of the study?	
Gender	4	Was the researcher male or female?	
Experience and training	5	What experience or training did the researcher have?	
Relationship with participants			
Relationship established	6	Was a relationship established prior to study commencement?	
Participant knowledge of the interviewer	7	What did the participants know about the researcher? e.g. personal goals, reasons for doing the research	
Interviewer characteristics	8	What characteristics were reported about the inter viewer/facilitator? e.g. Bias, assumptions, reasons and interests in the research topic	
**Domain 2: Study design**			
Theoretical framework			
Methodological orientation and Theory	9	What methodological orientation was stated to underpin the study? e.g. grounded theory, discourse analysis, ethnography, phenomenology, content analysis	
Participant selection			
Sampling	10	How were participants selected? e.g. purposive, convenience, consecutive, snowball	
Method of approach	11	How were participants approached? e.g. face-to-face, telephone, mail, email	
Sample size	12	How many participants were in the study?	
Non-participation	13	How many people refused to participate or dropped out? Reasons?	
Setting			
Setting of data collection	14	Where was the data collected? e.g. home, clinic, workplace	
Presence of non-participants	15	Was anyone else present besides the participants and researchers?	
Description of sample	16	What are the important characteristics of the sample? e.g. demographic data, date	
Data collection			
Interview guide	17	Were questions, prompts, guides provided by the authors? Was it pilot tested?	
Repeat interviews	18	Were repeat inter views carried out? If yes, how many?	
Audio/visual recording	19	Did the research use audio or visual recording to collect the data?	
Field notes	20	Were field notes made during and/or after the inter view or focus group?	
Duration	21	What was the duration of the inter views or focus group?	
Data saturation	22	Was data saturation discussed?	
Transcripts returned	23	Were transcripts returned to participants for comment and/or	

For peer review only

Topic	Item No.	Guide Questions/Description	Reported on Page No.
		correction?	
**Domain 3: analysis and findings**			
Data analysis			
Number of data coders	24	How many data coders coded the data?	
Description of the coding tree	25	Did authors provide a description of the coding tree?	
Derivation of themes	26	Were themes identified in advance or derived from the data?	
Software	27	What software, if applicable, was used to manage the data?	
Participant checking	28	Did participants provide feedback on the findings?	
Reporting			
Quotations presented	29	Were participant quotations presented to illustrate the themes/findings? Was each quotation identified? e.g. participant number	
Data and findings consistent	30	Was there consistency between the data presented and the findings?	
Clarity of major themes	31	Were major themes clearly presented in the findings?	
Clarity of minor themes	32	Is there a description of diverse cases or discussion of minor themes?	

Developed from: Tong A, Sainsbury P, Craig J. Consolidated criteria for reporting qualitative research (COREQ): a 32-item checklist for interviews and focus groups. *International Journal for Quality in Health Care*. 2007. Volume 19, Number 6: pp. 349 – 357

Once you have completed this checklist, please save a copy and upload it as part of your submission. DO NOT include this checklist as part of the main manuscript document. It must be uploaded as a separate file.

For peer review only

GRIPP2 short form

Section and topic	Item	Reported on page No
1: Aim	Report the aim of PPI in the study	4-5
2: Methods	Provide a clear description of the methods used for PPI in the study	4-5,6,8-15
3: Study results	Outcomes—Report the results of PPI in the study, including both positive and negative outcomes	15-17
4: Discussion and conclusions	Outcomes—Comment on the extent to which PPI influenced the study overall. Describe positive and negative effects	15-17
5: Reflections/critical perspective	Comment critically on the study, reflecting on the things that went well and those that did not, so others can learn from this experience	15-17

PPI: patient and public involvement.

From: Staniszewska, S., Brett, J., Simera, I. *et al.* GRIPP2 reporting checklists: tools to improve reporting of patient and public involvement in research. *Res Involv Engagem* **3,** 13 (2017). https://doi.org/10.1186/s40900-017-0062-2

For peer review only
